# Effects on mortality of different blood purification techniques in sepsis patients: an umbrella review of systematic reviews and meta-analyses

**DOI:** 10.1080/0886022X.2026.2698155

**Published:** 2026-07-16

**Authors:** Yue Yuan, Siyuan Lei, Aoyi Duan, Jiansheng Li

**Affiliations:** a Lung Disease Diagnosis and Treatment Center, National Medical Center, The First Affiliated Hospital of Henan University of Chinese Medicine, Zhengzhou, China; b Collaborative Innovation Center for Chinese Medicine and Respiratory Diseases Co-constructed by Henan Province & Education Ministry of P.R. China/Henan Key Laboratory of Chinese Medicine for Respiratory Diseases, Henan University of Chinese Medicine, Zhengzhou, China

**Keywords:** Sepsis, blood purification, umbrella review, systematic review, meta-analysis

## Abstract

Sepsis remains a leading cause of mortality worldwide, and extracorporeal blood purification has been widely implemented despite ongoing uncertainty regarding its survival benefit. We conducted an umbrella review of 42 systematic reviews and meta-analyses evaluating mortality across major extracorporeal blood purification modalities. Mortality estimates generally favored extracorporeal blood purification over conventional care, but effect sizes and consistency varied substantially by modality, and the overall certainty of evidence for mortality was low. At the review level, polymyxin B hemoperfusion showed a relatively consistent direction toward lower mortality, whereas renal replacement therapy–based strategies were typically closer to the null. However, most included reviews were of low or critically low methodological quality, with downgrading driven by risk of bias, inconsistency, and potential publication bias. Therefore, these findings should be interpreted cautiously and should not be considered definitive evidence of survival benefit. They highlight persistent evidentiary fragility and underscore the need for rigorously designed, phenotype-informed trials to clarify modality-specific effects in sepsis.

## KEY MESSAGES

**What is known:** Extracorporeal blood purification has been widely used as an adjunctive therapy for sepsis, but its mortality benefit remains uncertain across different modalities.

**What this study adds:** This umbrella review synthesizes evidence from 42 systematic reviews and meta-analyses and shows that mortality estimates generally favor extracorporeal blood purification, although effects vary substantially by modality and the overall certainty of evidence remains low.

**Potential impact:** These findings support cautious, modality- and phenotype-informed interpretation of extracorporeal blood purification in sepsis and highlight the need for rigorously designed trials with standardized protocols.

## GRAPHIC ABSTRACT

**Figure:**
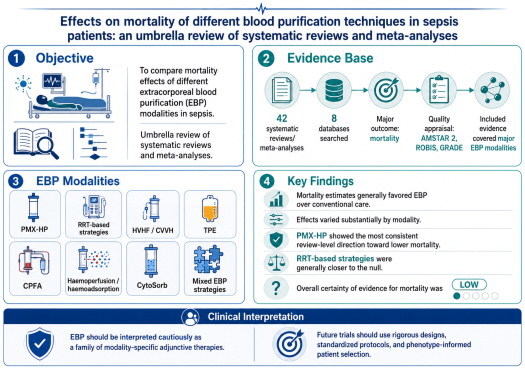

## Social media hook

An umbrella review of 42 systematic reviews suggests that mortality estimates generally favor extracorporeal blood purification in sepsis, but benefits vary by modality and the certainty of evidence remains low.

## 1. Introduction

Sepsis is a life-threatening syndrome in which infection triggers a dysregulated host response, resulting in acute organ dysfunction and substantial morbidity and mortality [1,2]. Despite advances in early recognition, antimicrobial therapy, source control, and contemporary intensive care, sepsis remains a major driver of preventable deaths worldwide [3]. It also places a considerable burden on health systems. The clinical course is highly heterogeneous, encompassing hyperinflammation, immune dysfunction, endothelial injury, microcirculatory impairment, and metabolic derangements – pathobiological features that contribute to refractory shock, progressive organ failure, and death [4].

These mechanistic considerations have prompted growing interest in adjunctive extracorporeal blood purification (EBP) therapies as a means to modulate circulating mediators involved in the pathophysiology of sepsis [5]. By facilitating the removal of endotoxin, inflammatory cytokines, and other immune-active molecules from the circulation, EBP is hypothesized to attenuate dysregulated host responses and potentially mitigate downstream organ dysfunction [6]. Beyond simple mediator removal, emerging mechanistic evidence suggests that extracorporeal therapies may also exert immunomodulatory effects by altering leukocyte activation states, modulating monocyte/macrophage and neutrophil responses, and influencing endothelial–immune and coagulation–inflammation interactions [7]. These effects may contribute to partial reprogramming of specific immune-cell subsets, although their translation into consistent clinical benefit likely depends on patient phenotype, timing of initiation, and the dominant immune state. In clinical practice, EBP has been applied using a variety of extracorporeal techniques, most commonly renal replacement therapy (RRT) and hemoperfusion approaches such as polymyxin B hemoperfusion (PMX-HP) [8–11]. However, despite widespread clinical use and biological plausibility, the extent to which EBP improves clinically meaningful outcomes, particularly mortality, remains uncertain.

Reported effects vary across trials and patient populations, likely reflecting the marked clinical and biological heterogeneity of sepsis [12]. Treatment effects may therefore depend on both modality characteristics and patient selection, as well as treatment timing and intensity. Interpretation is further complicated by methodological heterogeneity across primary studies and secondary syntheses, including differences in eligibility criteria, co-interventions, mortality time points, and analytical choices, as well as overlap of component trials across reviews [13].

Umbrella reviews provide a structured approach to synthesizing evidence across multiple reviews, particularly when the evidence base is rapidly expanding and conclusions are inconsistent [14]. In addition to summarizing effect estimates, umbrella reviews can appraise methodological quality and risk of bias across reviews, evaluate the certainty of evidence, and transparently characterize the robustness of conclusions [15,16]. They are also well suited to identifying evidence gaps, clarifying sources of discordance among reviews, and providing decision-relevant evidence to inform practice and guide future research [17].

However, no umbrella review has comprehensively evaluated blood purification strategies for sepsis with mortality as the primary outcome. Given the growing number of systematic reviews and meta-analyses and ongoing uncertainty regarding survival benefit, a rigorous umbrella synthesis is timely. We therefore conducted this umbrella review to synthesize the evidence on blood purification and mortality in sepsis, compare findings across extracorporeal modalities, and clarify the strength and consistency of the evidence base.

## 2. Methods

### 2.1. Study design

This systematic review of systematic reviews and meta-analyses (hereafter referred to as an umbrella review) was reported according to the Preferred Reporting Items for Systematic Reviews and Meta-Analyses (PRISMA) guidelines (S1 File) and was prospectively registered on PROSPERO on 1 March 2026 (CRD420261283626) [18].

### 2.2. Search strategy

Search strings were developed to capture concepts related to sepsis, blood purification/extracorporeal blood purification interventions, and systematic reviews/meta-analyses. The full search strategies and keywords are provided in the Supporting Information (S2 File). Eight databases were searched from inception to 1 May 2026, including four English-language databases (PubMed, Embase, Web of Science, and the Cochrane Library) and four Chinese databases (China National Knowledge Infrastructure, Wanfang Data, China Science and Technology Journal Database, and Chinese Biomedical Literature Database). Searches were restricted to studies published in English or Chinese, and, where applicable, were limited to human studies.

### 2.3. Eligibility criteria

Eligibility criteria were prespecified using the PICOS framework. We included systematic reviews of experimental studies enrolling patients of any age with sepsis or septic shock, evaluating extracorporeal blood purification strategies, including RRT, continuous venovenous hemofiltration (CVVH), continuous venovenous hemodiafiltration (CVVHDF), high-volume hemofiltration (HVHF), pulsed high-volume hemofiltration (PHVHF), hemoperfusion (including PMX-HP), hemoadsorption devices, therapeutic plasma exchange (TPE), and coupled plasma filtration adsorption (CPFA), CytoSorb, compared with usual care or standard therapy without blood purification. The primary outcome was mortality, defined as ICU, in-hospital, 28-day, or the longest reported follow-up mortality. Reviews were eligible whether they reported meta-analytic synthesis, narrative synthesis, or both, and were excluded if they involved non-human or non-sepsis populations, mixed critical-care populations without extractable sepsis-specific data, interventions used solely for monitoring or supportive purposes without an intended blood purification effect, comparisons that precluded a meaningful contrast because blood purification was applied in both arms, or outcomes that did not report mortality and could not be derived; we also excluded scoping or nonsystematic reviews, methodological papers, and primary studies, as well as records not published in peer-reviewed journals or indexed databases and conference abstracts without a full-text report (full-length conference articles were eligible). To improve clinical interpretability, the main mechanisms of the extracorporeal blood purification modalities considered in this review are summarized in Table 1.

**Table 1.** Mechanisms of extracorporeal blood purification modalities in sepsis.

Modality	Main mechanism/target
RRT	Provides solute and fluid removal; may allow partial clearance of small and middle molecules depending on membrane and dose
CVVH	Continuous convective solute and fluid removal through hemofiltration
CVVHDF	Combines diffusion and convection for continuous solute and fluid removal
HVHF	High-volume convective clearance intended to enhance removal of inflammatory mediators
PMX-HP	Adsorbs circulating endotoxin using polymyxin B–immobilized fibers
Hemoperfusion	Uses adsorptive cartridges to remove inflammatory mediators, endotoxin-related factors, or middle-to-large molecules
Hemoadsorption devices	Adsorptive removal of cytokines and other circulating mediators using device-specific cartridges or membranes
CytoSorb	Broad-spectrum hemoadsorption of cytokines and other hydrophobic molecules
TPE	Exchanges plasma to remove circulating mediators, endotoxin-related factors, and dysregulated plasma components while replacing plasma factors
CPFA	Separates plasma, passes it through an adsorptive cartridge, and reinfuses treated plasma
oXiris	Modified AN69 membrane with combined renal support, endotoxin adsorption, and cytokine adsorption
iHSA	Immobilized human serum albumin adsorber designed to bind endotoxin and other albumin-binding toxic mediators
Alteco	Endotoxin adsorption device using immobilized synthetic peptides or binding matrices to remove circulating endotoxin.

### 2.4. Data extraction

Records retrieved from the database searches were imported into EndNote X9 and duplicates were removed. Titles, abstracts, and full texts were then screened within EndNote. Each record was independently assessed by two reviewers (Yue Yuan and Aoyi Duan), and any disagreements were resolved through discussion with a third reviewer (Siyuan Lei).

Data extraction was performed independently by two reviewers using a standardized, pilot-tested extraction form. Extracted items included bibliographic information (first author and publication year), key characteristics of each systematic review (type of synthesis, number of included primary studies, total sample size, and population/setting), details of blood purification interventions (e.g., modality and treatment strategy), comparators, and outcome data. For mortality outcomes, we preferentially extracted the effect estimates reported in each review including risk ratios(RR) with 95% confidence intervals (CI). When a single systematic review reported multiple mortality estimates corresponding to different blood purification modalities, each estimate was extracted and retained separately.

### 2.5. Statistical analysis

We assessed the degree of overlap of component primary studies (e.g., randomized controlled trials) across the included systematic reviews and meta-analyses. The overlap in component primary studies included in all eligible reviews was assessed using the Corrected Covered Area (CCA) formula: CCA =*(N − r)/(rc − r),* where *N* is the sum of total primary studies included in all the reviews, *r* is the number of unique primary studies, and *c* is the total number of reviews. Overlap was quantified using the CCA method, whereby a CCA of 100% indicates that all included reviews comprised exactly the same component studies, whereas a CCA of 0% indicates that the component studies were entirely unique across reviews. CCA values were interpreted as slight (0–5%), moderate (6–10%), high (11–15%), or very high (>15%) overlap.

Because overlap of component studies can lead to double-counting, a quantitative ‘meta-analysis of meta-analyses’ was not undertaken as the primary synthesis approach. Instead, we conducted a structured narrative synthesis. Meta-analyses were grouped by outcome (primary outcome: mortality) and summarized using forest plots that display, for each meta-analysis, the reported effect estimate (risk ratio with 95% CI), sample size (where available), and statistical heterogeneity. Effect estimates were presented as originally reported in each systematic review. When a single review reported multiple mortality estimates corresponding to different blood purification modalities, each estimate was extracted and displayed as a separate entry.

Where systematic reviews used narrative synthesis without pooled estimates, we summarized the direction and statistical significance of findings as reported by the review authors. Sensitivity analyses were undertaken descriptively to examine whether conclusions were consistent after excluding systematic reviews rated as low or critically low confidence by AMSTAR 2. In addition, we planned modality-based subgroup synthesis (e.g., RRT, hemoperfusion/PMX-HP, TPE, hemoadsorption, CPFA) to explore whether results differed by blood purification strategy.

### 2.6. Methodological quality assessment

The methodological quality of included systematic reviews was independently assessed by two reviewers using the AMSTAR 2 instrument, which includes 16 items. Each item was rated as ‘Yes’, ‘Partial Yes’, or ‘No’, and overall confidence was categorized as high, moderate, low, or critically low in accordance with AMSTAR 2 guidance. In AMSTAR 2, high and moderate ratings indicate greater methodological confidence, whereas low and critically low ratings indicate reduced methodological confidence.

The risk of bias of included systematic reviews was evaluated using the ROBIS tool across Phase 1 (relevance), Phase 2 (four domains: study eligibility criteria; identification and selection of studies; data collection and study appraisal; synthesis and findings), and Phase 3 (overall risk of bias). Each ROBIS domain and the overall judgment were rated as low, high, or unclear risk of bias. In ROBIS, low risk indicates a more favorable risk-of-bias judgment, whereas high risk indicates greater concern for bias. Disagreements between the two reviewers were resolved through discussion; if consensus could not be reached, a third reviewer adjudicated.

### 2.7. Assessment of the certainty of evidence

The certainty of the evidence for the primary outcome (mortality) was assessed using the GRADE (Grading of Recommendations Assessment, Development and Evaluation) framework. Evidence certainty was evaluated across five domains that may warrant downgrading: risk of bias, inconsistency, indirectness, imprecision, and publication bias. Upgrading was considered in the presence of a large magnitude of effect, a dose–response gradient, or when all plausible residual confounding would likely reduce an observed effect. The overall certainty of evidence was categorized as high, moderate, low, or very low. In GRADE, high and moderate certainty ratings indicate greater confidence in the estimated effect, whereas low and very low certainty ratings indicate reduced confidence. GRADE assessments were performed independently by two reviewers, with discrepancies resolved by discussion or adjudication by a third reviewer.

## 3. Results

### 3.1. Results of the search

**Figure 1.**
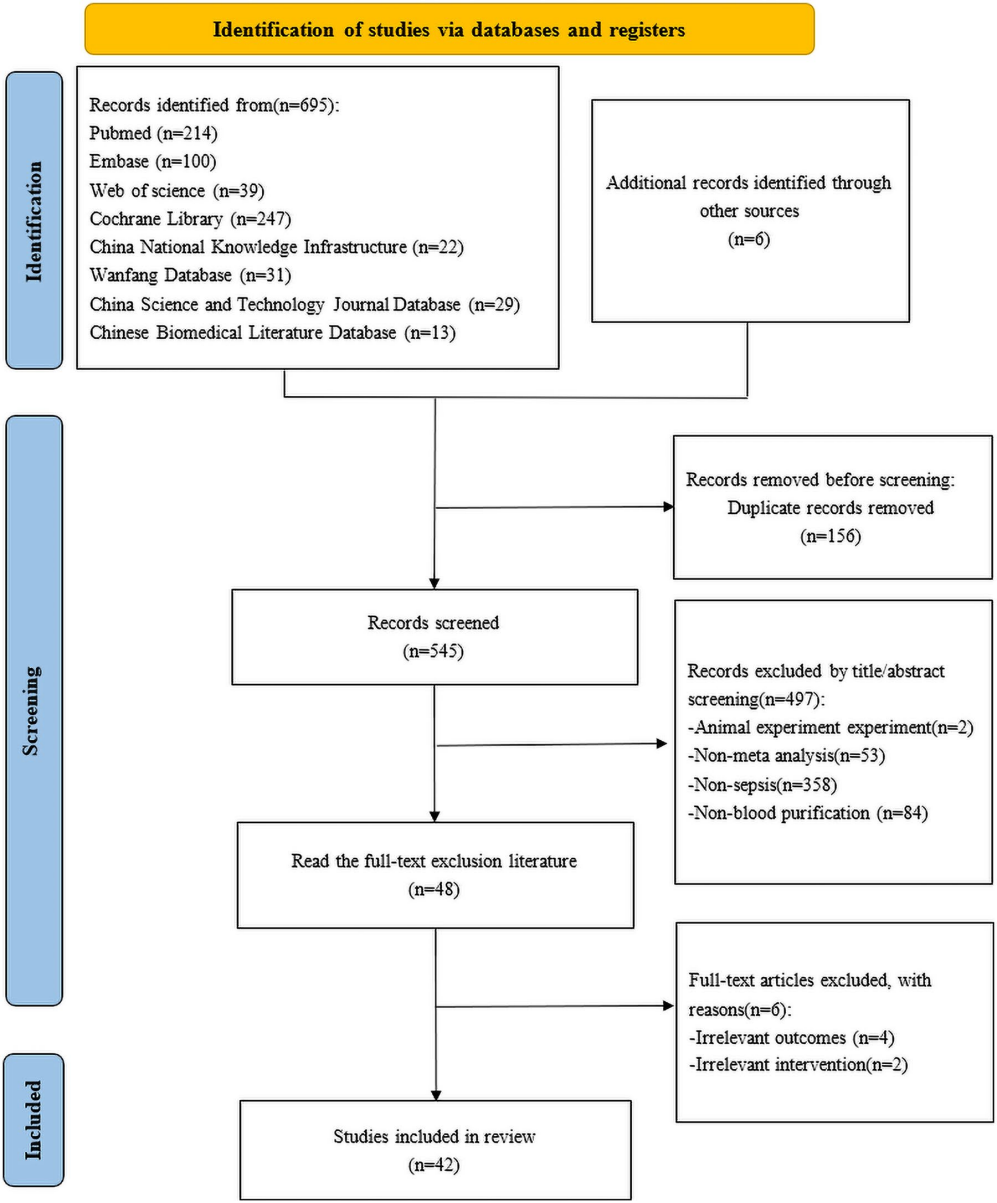
Flow diagram of study inclusion.

The search strategy initially yielded 701 studies. After removing duplicates, 545 studies remained. Title and abstract screening excluded 497 studies, leaving 48 for full-text review to determine their suitability for inclusion. Ultimately, 42 studies met the criteria and were incorporated into the analysis. This literature selection process is detailed in Figure 1.

### 3.2. Study characteristics

Table 2 summarizes the key characteristics of the 42 included systematic reviews and meta-analyses, including 39 conventional systematic reviews/meta-analyses and 3 network meta-analyses [19–59]. The included reviews were published between 2007 and 2026, with a clear concentration of publications in more recent years. Across reviews, the number of primary studies included per review ranged from 4 to 60, and sample sizes varied widely, from 194 to 28,291 participants. Most reviews synthesized evidence from randomized controlled trials, although several also included cohort or other controlled study designs. The evaluated extracorporeal blood purification strategies were heterogeneous, spanning PMX-HP, hemoperfusion techniques, hemofiltration approaches, continuous blood purification or extracorporeal blood purification, RRT, TPE, CPFA, hemoadsorption devices, and network comparisons across multiple modalities. Outcomes most frequently included mortality, including all-cause, in-hospital, hospital, overall, and 28-day mortality, alongside organ dysfunction and severity scores such as the Sequential Organ Failure Assessment (SOFA) and the Acute Physiology and Chronic Health Evaluation II (APACHE II), hemodynamic parameters, inflammatory biomarkers/cytokines, and resource-use endpoints such as ICU length of stay (LOS). Detailed mortality effect estimates, including the reported mortality time point, RR, and 95% CI for each included review, are provided in S3 File.

**Table 2.** Summary of findings of the included meta-analyses.

Review type	Study (Author, Year)	No. of studies	Sample size	Age (years)	Study design	Databases searched	Search date	Effect model	Blood purification modality	Outcomes	Main findings
Meta-analysis	Dinna et al. 2007 [19]	28	1,425	39–78.5	RCTs, cohort studies	PubMed, Cochrane	April 2006	Random-effects	PMX-HP	MAP, vasopressor dose, PaO₂/FiO₂, endotoxin levels, mortality	PMX hemoperfusion was associated with improved mean arterial pressure, reduced vasopressor requirements, enhanced oxygenation, endotoxin removal, and reduced mortality.
	Liu et al. 2010 [20]	9	337	56–70	RCTs, cohort studies	CNKI, CBM, VIP, Wanfang, PubMed, MEDLINE, EMBASE, Web of Science	January 1999–December 2009	Fixed-effect	HVHF	Mortality	HVHF was associated with reduced mortality in patients with septic shock.
	Latour-Pérez et al. 2011 [21]	12	505	≥16	RCTs	PubMed, EMBASE, Cochrane, ClinicalTrials.gov, CNKI	January 1999–December 2009	Random-effects	CVVH	Hemodynamics, lung function, MODS, mortality	Current evidence does not support routine use of RRT in sepsis.
	Zhou et al. 2011 [22]	16	827	≥18	RCTs	PubMed, EMBASE, Cochrane	1 July 2011	Fixed-effect	HP + TPE	All-cause mortality	Blood purification techniques may reduce mortality in sepsis or septic shock.
	Tian et al. 2012 [23]	11	414	≥18	RCTs	PubMed, Cochrane, EMBASE, CNKI	January 2011	Fixed-effect	BP	Mortality, LOS, IL-6, TNF-α, WBC, hemodynamics	Continuous blood purification did not reduce mortality but effectively removed inflammatory mediators and shortened hospital stay.
	Zhou et al. 2014 [24]	16	827	≥18	RCTs	MEDLINE, EMBASE, Cochrane	1 January 1966–1 May 2012	Random-effects	HP; PHVHF; CVVH; CVVHDF; TPE	APACHE II/, SAPS II, SOFA	Blood purification was associated with lower mortality and improved severity scores.
	Clark et al. 2014 [25]	4	466	≥18	RCTs	MEDLINE, EMBASE, Cochrane	January 1966–December 2013	Random-effects	HVHF	Mortality, vasopressor dose, renal recovery	Evidence was insufficient to support routine HVHF use outside experimental settings.
	Rimmer et al. 2014 [26]	4	194	—	RCTs	PubMed, EMBASE, Cochrane, conference proceedings	28 April 2014	Random-effects	TPE	Mortality	Insufficient evidence to recommend TPE as adjunctive therapy for sepsis or septic shock.
	Chen et al. 2015 [27]	6	1,295	≥18	RCTs	PubMed, Cochrane, EMBASE, CNKI, VIP, Wanfang	July 2014	Fixed-effect	HVHF	28-day mortality	HVHF was not superior to SVHF and was associated with more adverse events.
	Gong et al. 2015 [28]	5	885	—	Controlled studies	PubMed, Web of Science, EMBASE, CNKI, VIP	January 2000–July 2014	Random-effects	RRT	Mortality, ICU LOS	Early RRT did not reduce overall mortality but was associated with lower 28-day mortality.
	Putzu et al. 2017 [29]	11	679	—	RCTs	PubMed, EMBASE, CENTRAL	1 March 2017	Random-effects	CVVH	Short- and long-term mortality, ICU LOS	Low-quality evidence suggests CVVH may reduce mortality in sepsis or ARDS.
	Zhen et al. 2017 [30]	6	275	≥18	RCTs	PubMed, Cochrane, CNKI, Wanfang	27 January 2017	Fixed-effect	HP	ICU LOS, ventilation duration, MAP, IL-6, mortality	HA330 hemoperfusion improved hemodynamics and oxygenation and reduced mortality.
	Chang et al. 2017 [31]	17	—	≥18	RCTs, cohort studies	PubMed, EMBASE, Cochrane	May 2016	Random-effects	PMX-HP	Mortality	PMX-HP may reduce mortality in selected high-severity subgroups.
	Terayama et al. 2017 [32]	7	841	≥18	RCTs	CENTRAL, MEDLINE, Scopus, SCI	February 2015	—	PMX-HP	APACHE II, mortality	PMX-DHP was associated with reduced mortality in sepsis and septic shock.
	Huang et al. 2018 [33]	11	410	≥18	RCTs	MEDLINE, EMBASE, Cochrane, CNKI	25 March 2017	Fixed-effect	PHVHF	Mortality, cytokines, APACHE II	PHVHF improved prognosis compared with standard therapy.
	Kuriyama et al. 2018 [34]	7	586	—	RCTs	PubMed, EMBASE, Cochrane, CINAHL	30 July 2017	Fixed-effect	PMX-HP	28-day mortality, adverse events	No evidence supporting PMX-HP for improving 28-day mortality.
	Putzu et al. 2019 [35]	37	2,499	≥18	RCTs	PubMed, Cochrane, EMBASE	1 January 2019	—	HP; RRT; TPE	Mortality, APACHE II, SOFA	Blood purification modalities may reduce mortality in sepsis or septic shock.
	Li et al. 2019 [36]	5	900	≥18	RCTs	PubMed, Cochrane, EMBASE	31 March 2019	Random-effects	RRT	28-day, 90-day mortality	Early RRT initiation did not reduce mortality in septic AKI.
	Snow et al. 2019 [37]	39	2,729	—	RCTs	MEDLINE, EMBASE, Cochrane, ClinicalTrials.gov	—	—	HAD; RRT; PMX-HP	Mortality	Endotoxin removal devices were associated with reduced mortality.
	Zayed et al. 2019 [38]	4	936	—	RCTs	PubMed, EMBASE, Cochrane	15 October 2018	—	RRT	Mortality, ICU LOS	Early RRT initiation was associated with reduced mortality.
	Tian et al. 2020 [39]	6	471	—	RCTs	PubMed, EMBASE, Cochrane	January 1998–October 2018	Random-effects	PMX-HP	28-day mortality	PMX-HP did not improve outcomes in sepsis or septic shock.
	Yin et al. 2020 [40]	5	241	≥18	RCTs	PubMed, EMBASE, Cochrane	21 June 2019	—	RRT; HVHF; CVVH	28-day mortality	HVHF-containing strategies did not improve outcomes.
	Li et al. 2021 [41]	13	1,163	—	RCTs	PubMed, EMBASE, Cochrane, CENTRAL	April 2020	—	PMX-HP	Mortality	PMX-HP reduced mortality in less severe sepsis and appeared safe.
	Snow et al. 2021 [37]	39	2,729	≥16	RCTs	PubMed, EMBASE, Cochrane, CENTRAL	December 2019	—	CVVH; HAD; CPFA; TPE	Mortality	Evidence remains insufficient to support routine extracorporeal blood purification.
	Xiao et al. 2022 [42]	28	2,587	—	RCTs	PubMed, Web of Science, Cochrane, CNKI	January 2010–January 2020	Random-effects	BP	In-hospital mortality, ICU LOS	Extracorporeal blood purification may be considered as adjunctive therapy.
	Li et al. 2022 [43]	6	537	≥14	RCTs, cohort studies	PubMed, EMBASE, Cochrane	1 May 2022	—	CPFA	All-cause mortality	CPFA did not reduce mortality in sepsis or septic shock.
	Mohammed et al. 2022 [44]	5	4,329	≥18	RCTs	PubMed, Ovid	January 2000–October 2020	—	RRT	Mortality, adverse events	Early initiation may not significantly affect mortality.
	Olive et al. 2023 [45]	14	632	—	RCTs, controlled studies	PubMed, EMBASE, CINAHL, Cochrane	1 December 2022	—	TPE	Mortality, organ dysfunction	TPE may benefit adults with severe sepsis but not children.
	Yan et al. 2023 [46]	13	1,263	—	RCTs	PubMed, MEDLINE, EMBASE, Cochrane	January 2017–July 2022	—	BP	Mortality, ICU LOS	Adjunctive blood purification reduced mortality and ICU LOS.
	Szigetváry et al. 2023 [46]	26	243	—	RCTs, controlled studies	PubMed, EMBASE, CENTRAL, Scopus	17 December 2021	—	HA	Oxygenation, biomarkers, mortality	Hemoadsorption improved oxygenation and inflammatory profiles.
	Zhang et al. 2023 [48]	5	390	—	RCTs	PubMed, EMBASE, Cochrane, CENTRAL	31 October 2021	Random-effects	TPE	Mortality, LOS	Plasma exchange appeared effective in reducing mortality.
	Jiovany et al. 2023 [54]	6	413	≥18	RCTs	PubMed, Embase, Cochrane	November 2022	Random-effects	CytoSorb	Mortality at 28–30 days, vasopressors, inflammatory marker, adverse events	Thereby showing no benefit of CytoSorb use in terms of mortality at 28–30 days.
	Wu et al. 2024 [49]	19	647	—	RCTs	PubMed, Web of Science, MEDLINE, CNKI	30 June 2023	Fixed-effect	HP	Cytokines, SOFA, mortality	Combined therapy reduced inflammatory mediators and mortality.
	Wang et al. 2024 [50]	12	3,648	—	RCTs	PubMed, Web of Science, Cochrane	28 March 2022	Fixed-effect	RRT	28-day mortality	Early RRT improved short-term survival in selected patients.
	Hernandez et al. 2024 [51]	6	283	—	RCTs, cohort studies	PubMed, Cochrane, CENTRAL	6 September 2023–5 January 2024	Fixed-effect	TPE	Mortality	Strong evidence supports TPE in septic shock.
	Kuklin et al. 2024 [52]	20	937	—	RCTs, cohort studies	PubMed, EMBASE, Cochrane	1 January 1966–1 November 2022	Random-effects	TPE	Mortality	Adjunctive TPE may confer survival benefit.
	Li et al. 2024 [53]	35	28,291	≥18	RCTs, cohort studies	PubMed, EMBASE, Cochrane	30 June 2023	Random-effects	PMX-HP	All-cause mortality	PMX-HP reduced 28-day mortality in sepsis or septic shock.
	Steindl et al. 2025 [55]	9	744	—	RCT, observational studies	PubMed, Cochrane	10 June 2024	Random-effects	CytoSorb	Mortality, ICU and hospital length of stay, the duration of mechanical ventilation, vasopressor requirements, lactate levels, SOFA scores	The use of CytoSorb alongside standard of care management may be linked to improved short-term survival in patients with septic shock
	Orban et al. 2025 [56]	10	715	—	RCTs, case–control studies	PubMed, EMBASE, Scopus, Web of Science	2016 to September 2024	Random-effects	CytoSorb	Mortality, ICU Length of Stay and Severity Scores	Current evidence does not support the routine use of CytoSorb in critically ill septic patients.
Network meta-analyse	Chen et al. 2023 [57]	60	4,458	—	RCTs	PubMed, EMBASE, Cochrane, MEDLINE	26 September 2022	—	Alteco LPS Adsorber, CPFA, CytoSorb, HA, iHSA, IL, Plasma exchange, PMX-HP, CVVH, Selective cytopheretic device	Overall mortality	Plasma exchange and PMX-HP may provide potential benefits for adult patients with severe infection or sepsis/septic shock.
	Xing et al. 2025 [58]	11	941	—	RCTs	PubMed, EMBASE, Cochrane, Web of Science	14 October 2024	Fixed-effect	HA, PMXHP, CPFA, oXiris, Cytosorb, CRRT, HP	Hospital mortality	HA and PMX showed superior overall efficacy in sepsis patients compared with other modalities.
	Meco et al. 2026 [59]	31	379	—	RCTs	English databases	December 2024	Fixed-effect	ALT cartridge, PMX, CPFA, HA, Cytosorb cartridge, oXiris, iHSA, PE, CRRT, CVVH.	Hospital mortality	The PMX filter and HA 330 for blood purification greatly lower mortality rates in patients experiencing severe septic shock.

Note: BP, blood purification; RRT, renal replacement therapy; CVVH, continuous venovenous hemofiltration; CVVHDF, continuous venovenous hemodiafiltration; HVHF, high-volume hemofiltration; PHVHF, pulsed high-volume hemofiltration; HP, hemoperfusion; PMX-HP, polymyxin B hemoperfusion; HA, hemoadsorption; TPE, therapeutic plasma exchange; CPFA, coupled plasma filtration adsorption; OEAHP, other endotoxin adsorption hemoperfusion; ALT, Alteco; EFF, Efferon; HVCVVH, high volume continuous veno-venous hemofiltration; VHVCVVH, very high volume continuous veno-venous hemofiltration; iHSA, immobilized human serum albumin; IH, intermittent hemodialysis; AKI, acute kidney injury; ARDS, acute respiratory distress syndrome; MODS, multiple organ dysfunction syndrome.

To account for temporal changes in sepsis definitions, we descriptively classified the included reviews according to the sepsis-definition context in which they were published. Reviews were grouped into three evidence periods: a SIRS-based/pre-Sepsis-2 period, reflecting the influence of the 1991/1992 ACCP/SCCM consensus framework; a Sepsis-2–dominant transition period, reflecting continued use of the 2001 International Sepsis Definitions Conference concepts of sepsis, severe sepsis, septic shock, and organ dysfunction; and a Sepsis-3–influenced contemporary period, reflecting the increasing adoption of the 2016 Sepsis-3 definition emphasizing life-threatening organ dysfunction caused by a dysregulated host response to infection. This classification was descriptive and based on the publication context of each review rather than an assumption that all primary studies within a given review used the same diagnostic definition. The temporal stratification of the included reviews is summarized in Table 3.

**Table 3.** The temporal stratification of the included reviews.

Evidence period	Publication years	Sepsis-definition context	Dominant blood purification research context	Included reviews
Pre-Sepsis-2 / SIRS-based consensus period	2007–2012	Mainly influenced by the ACCP/SCCM SIRS-based framework and early severe sepsis/septic shock definitions	Early PMX-HP, HVHF, CVVH, and broadly defined blood purification strategies	Dinna 2007 [19]; Liu 2010 [20]; Latour-Pérez 2011 [29]; Zhou 2011 [24]; Tian 2012 [23]
Sepsis-2–dominant transition period	2013–2018	Continued use of sepsis, severe sepsis, septic shock, and organ dysfunction concepts from the 2001 definitions	Diversification of modalities, including HVHF, TPE, PMX-HP, CPFA, and hemoperfusion; increasing PMX-HP controversy	Zhou 2014 [22]; Clark 2014 [25]; Rimmer 2014 [26]; Chen 2015 [27]; Gong 2015 [28]; Putzu 2017 [35]; Zhen 2017 [30]; Chang 2017 [31]; Terayama 2017 [32]; Huang 2018 [33]; Kuriyama 2018 [34]
Sepsis-3–influenced contemporary period	2019–2026	Increasing influence of Sepsis-3, emphasizing life-threatening organ dysfunction caused by dysregulated host response to infection	RRT timing, adsorption technologies, TPE, PMX-HP reassessment, and NMA-informed comparisons	Putzu 2019 [35]; Li 2019 [36]; Snow 2019 [37]; Zayed 2019 [38]; Tian 2020 [39]; Yin 2020 [40]; Li 2021 [43]; Snow 2021 [37]; Xiao 2022 [42]; Li 2022 [43]; Mohammed 2022 [44]; Olive 2023 [45]; Yan 2023 [40]; Szigetváry 2023 [47]; Zhang 2023 [48]; Jiovany 2023 [54]; Wu 2024 [49]; Wang 2024 [50]; Hernandez 2024 [51]; Kuklin 2024 [52]; Li 2024 [53]; Steindl 2025 [55]; Orban 2025 [56]; Chen et al. 2023 [57]; Xing et al. 2025 [58]; Meco 2026 [59]

### 3.3. Study overlap

Across the 42 included systematic reviews/meta-analyses, the total number of component primary-study occurrences reported across reviews was 632, of which 419 were unique after de-duplication. The corrected covered area (CCA) was 1.24%, indicating slight overlap in primary studies across reviews (S4 File). In a sensitivity calculation excluding network meta-analyses, the CCA was 1.31%, suggesting that inclusion of network meta-analyses did not materially change the degree of overlap.

### 3.4. Study quality assessment

**Figure 2.**
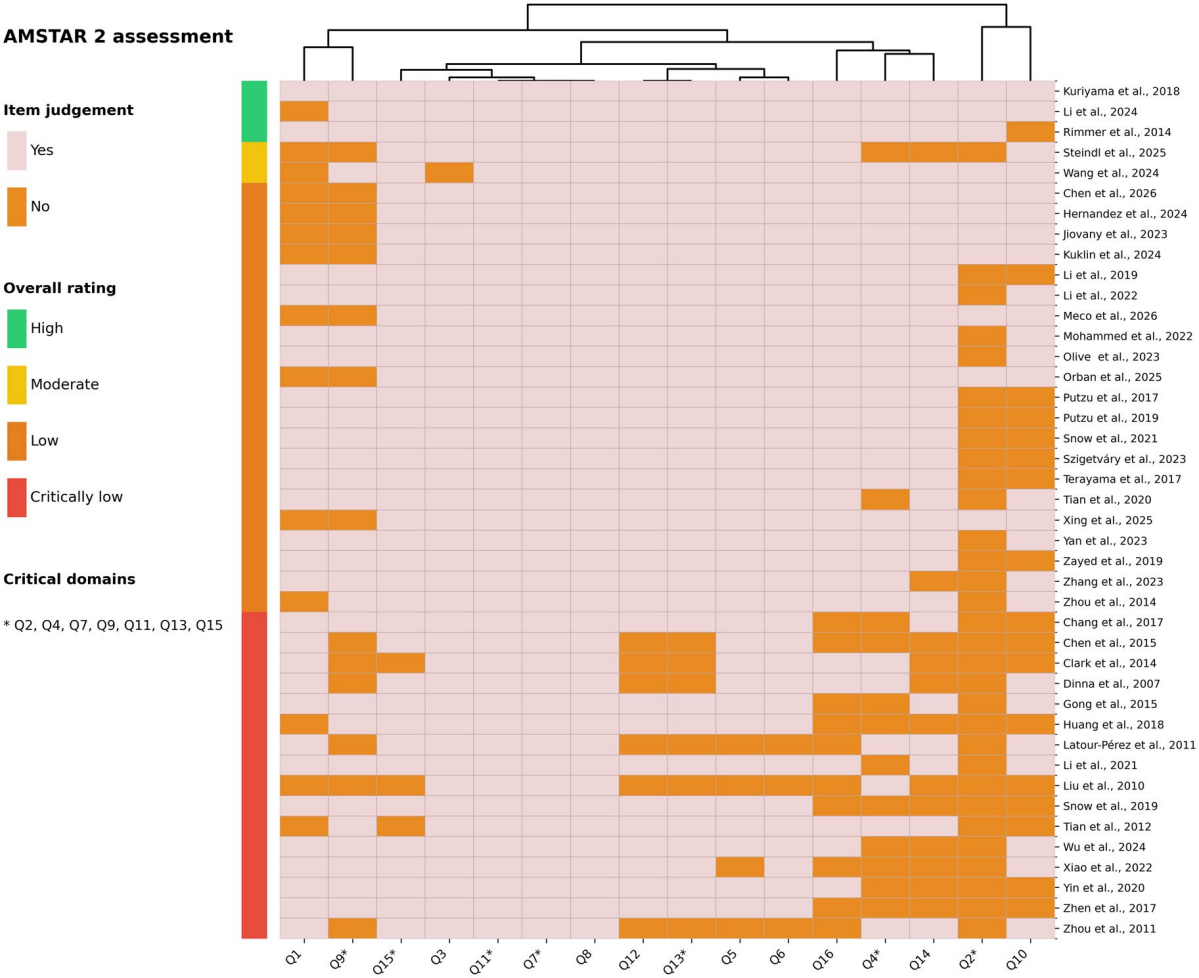
AMSTAR 2 assessment of included systematic review.

Using AMSTAR 2 (Figure 2), of the 42 included systematic reviews/meta-analyses, 3 (7.1%) were rated High, 2 (4.8%) Moderate, 21 (50.0%) Low, and 16 (38.1%) Critically low. Across the 16 AMSTAR 2 items, the most frequently unmet criteria were protocol registration/prior methods (Q2: 11/42 ‘Yes’), reporting funding sources of included primary studies (Q10: 25/42 ‘Yes’), comprehensive search strategy (Q4: 30/42 ‘Yes’), satisfactory explanation/discussion of heterogeneity (Q14: 30/42 ‘Yes’), and risk-of-bias assessment using a satisfactory technique (Q9: 28/42 ‘Yes’). Additional commonly missing elements included reporting potential conflicts of interest for the review (Q16: 32/42 ‘Yes’) and consideration of risk of bias when interpreting results (Q13: 36/42 ‘Yes’).

**Figure 3.**
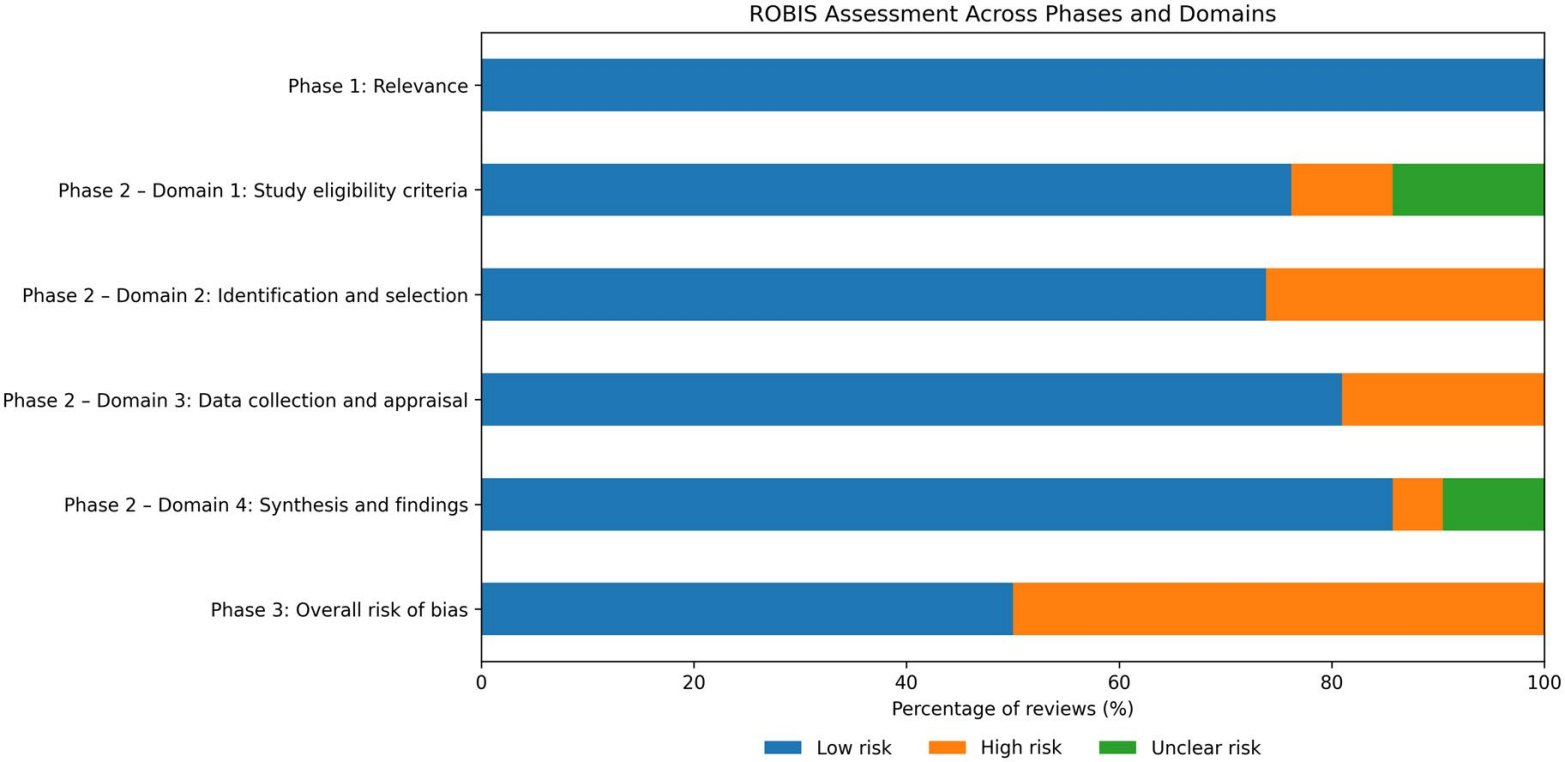
ROBIS assessment of included systematic review.

Based on the ROBIS tool (Figure 3), Phase 1 (Relevance) was judged Low risk for all 42 reviews (100%). In Phase 2, the distribution of judgments was: Domain 1 (Eligibility criteria)—Low 32 (76.2%), High 4 (9.5%), Unclear 6 (14.3%); Domain 2 (Identification and selection of studies)—Low 31 (73.8%), High 11 (26.2%); Domain 3 (Data collection and study appraisal)—Low 34 (81.0%), High 8 (19.0%); and Domain 4 (Synthesis and findings)—Low 36 (85.7%), High 2 (4.8%), Unclear 4 (9.5%). For Phase 3 (Overall risk of bias), 21 reviews (50.0%) were rated Low, and 21 (50.0%) were rated High.

For certainty of evidence, the GRADE assessment for the mortality outcome resulted in an overall rating of Low certainty, with downgrading driven by risk of bias, inconsistency, and publication bias, while indirectness and imprecision were not downgraded (S5 File).

### 3.5. Overall effects on mortality

**Figure 4.**
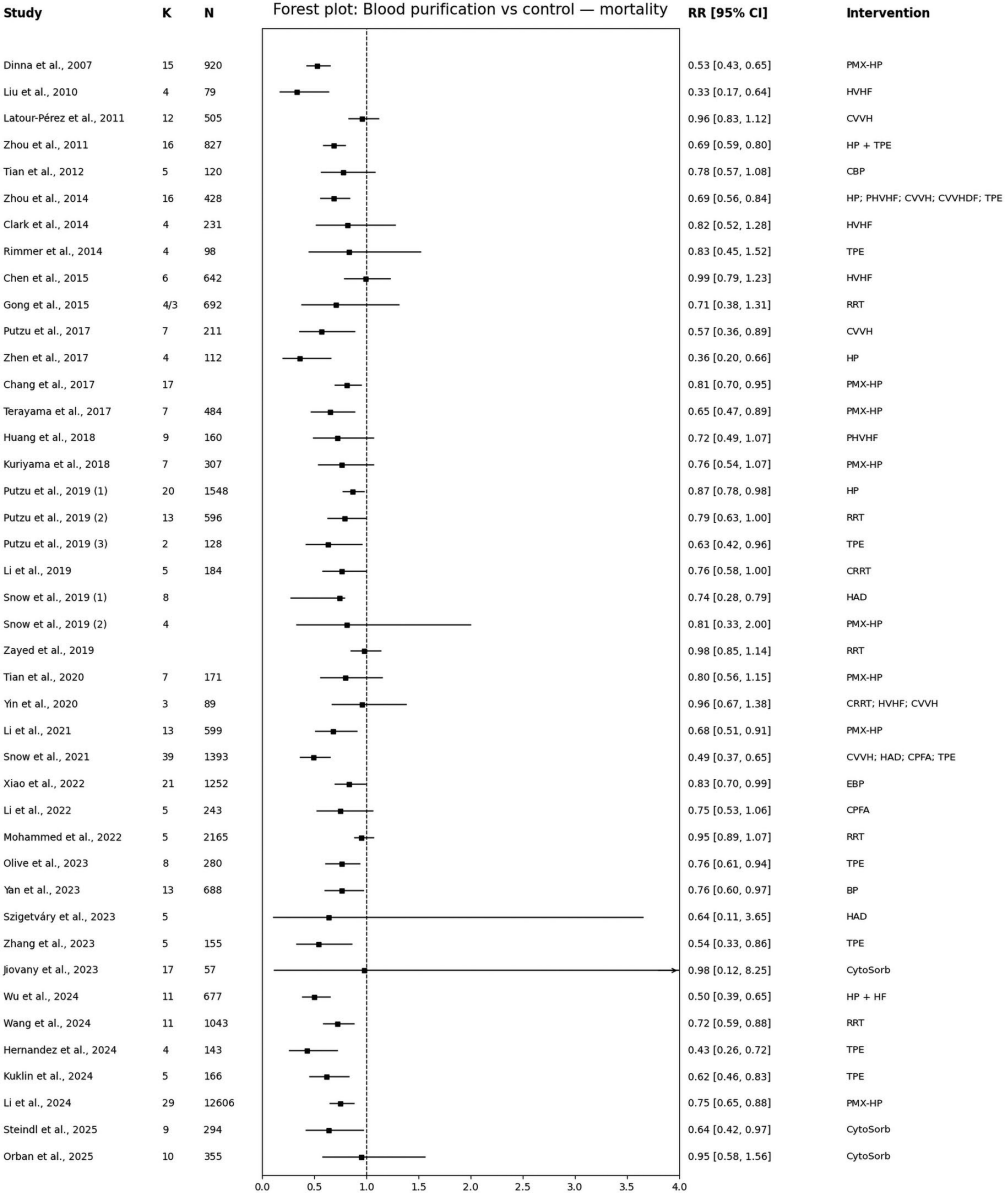
Summary of meta-analysis results for EBP interventions.

Across the included meta-analyses, the association between extracorporeal blood purification and mortality was generally in the direction of benefit but showed substantial variability in magnitude and statistical significance (Figure 4). Of the 40 mortality effect estimates extracted, 22 reported a statistically significant reduction in mortality, with RRs ranging from 0.33 to 0.87. The largest relative reductions were reported in Liu et al. [20] (RR 0.33, 95% CI 0.17–0.64), Hernandez et al. [51] (RR 0.43, 95% CI 0.26–0.72), Snow et al. [37] (RR 0.49, 95% CI 0.37–0.65), Wu et al. [49] (RR 0.50, 95% CI 0.39–0.65), and Li et al. [53] (RR 0.75, 95% CI 0.65–0.88). Several additional estimates also indicated benefit with narrower confidence intervals that did not cross the null (e.g., Zhou et al. [24] RR 0.69, 95% CI 0.59–0.80; Zhou et al. [22] RR 0.69, 95% CI 0.56–0.84; Putzu et al. [35] RR 0.57, 95% CI 0.36–0.89; Zhang et al. [48] RR 0.54, 95% CI 0.33–0.86).

In contrast, a considerable proportion of estimates were inconclusive, with confidence intervals overlapping 1.0, including Chen et al. [27] (RR 0.99, 95% CI 0.79–1.23), Latour-Pérez et al. [29] (RR 0.96, 95% CI 0.83–1.12), Yin et al. [40] (RR 0.96, 95% CI 0.67–1.38), and Mohammed et al. [44] (RR 0.95, 95% CI 0.89–1.07). Some estimates were accompanied by wide confidence intervals, suggesting imprecision and/or limited evidence (e.g., Szigetváry et al. [47] RR 0.64, 95% CI 0.11–3.65). Overall, the evidence landscape indicates potential mortality benefit of blood purification in sepsis, while also highlighting heterogeneity across reviews and blood purification approaches.

Each square represents the pooled RR reported by an included meta-analysis, and the horizontal line indicates the corresponding 95% CI. Meta-analyses with multiple blood purification modalities contributed multiple effect estimates, which are displayed as separate entries. The vertical dashed line denotes the line of no effect (RR = 1.0). An asterisk (*) indicates that the 95% CI does not include 1.0.

### 3.6. Effects across blood purification modalities

Across modality-specific analyses, the direction and magnitude of association between blood purification and mortality varied (S6 File). Because primary studies may overlap across reviews, the numbers of studies and patients reported below refer to the counts reported in the corresponding meta-analyses and should be interpreted descriptively rather than as de-duplicated unique counts. For unspecified blood purification, two meta-analyses suggested a reduction in mortality, Xiao et al. [42] reported RR 0.83, 95% CI 0.70–0.99 and Yan et al. [40] reported RR 0.76, 95% CI 0.60–0.97, whereas Tian et al. [23] showed no clear difference, RR 0.78, 95% CI 0.57–1.08. Overall, 2 reviews involving 34 reported studies and 1,940 patients showed favorable findings, while 1 review involving 5 studies and 120 patients did not demonstrate a clear effect.

For CVVH, evidence was inconsistent, with one meta-analysis indicating benefit, Putzu et al. [29], RR 0.57, 95% CI 0.36–0.89, while Latour-Pérez et al. [21] showed no clear effect, RR 0.96, 95% CI 0.83–1.12. Overall, 1 review involving 7 reported studies and 211 patients showed reduced mortality, while 1 review involving 12 studies and 505 patients did not demonstrate a clear effect.

For HAD devices, two meta-analyses suggested lower mortality, Zhen et al. [30], RR 0.36, 95% CI 0.20–0.66 and Snow et al., RR 0.74, 95% CI 0.28–0.79, whereas Szigetváry et al. [47] reported substantial imprecision with no clear effect, RR 0.64, 95% CI 0.11–3.65. Overall, 2 reviews involving 12 reported studies and at least 112 patients showed favorable findings, while 1 review involving 5 studies did not demonstrate a clear effect.

For HVHF/PHVHF, findings were mixed and largely non-significant except for one small meta-analysis, Liu et al. [20], RR 0.33, 95% CI 0.17–0.64; other syntheses did not demonstrate a clear mortality reduction, including Clark et al. [25], RR 0.82, 95% CI 0.52–1.28; Chen et al. [27], RR 0.99, 95% CI 0.79–1.23; and Huang et al. [33], RR 0.72, 95% CI 0.49–1.07. Overall, 1 review involving 4 reported studies and 79 patients showed reduced mortality, while 3 reviews involving 19 studies and 1,033 patients did not demonstrate a clear effect.

For PMX-HP, several meta-analyses consistently suggested lower mortality, including Dinna et al. [19], RR 0.53, 95% CI 0.43–0.65; Chang et al. [31], RR 0.81, 95% CI 0.70–0.95; Terayama et al. [32], RR 0.65, 95% CI 0.47–0.89; Li et al. [43], RR 0.68, 95% CI 0.51–0.91; and Li et al. [53], RR 0.75, 95% CI 0.65–0.88. In contrast, other PMX-HP syntheses were compatible with no effect, such as Kuriyama et al. [34], RR 0.76, 95% CI 0.54–1.07 and Tian et al. [39], RR 0.80, 95% CI 0.56–1.15, and one analysis was markedly imprecise, Snow et al. [37], RR 0.81, 95% CI 0.33–2.00. Overall, 5 reviews involving 81 reported studies and at least 14,609 patients showed favorable findings, while 3 reviews involving 18 studies and at least 478 patients did not demonstrate a clear effect.

For RRT, evidence was heterogeneous, with a significant reduction observed in Wang et al. [50], RR 0.72, 95% CI 0.59–0.88, whereas other meta-analyses did not show a clear difference, including Gong et al. [28], RR 0.71, 95% CI 0.38–1.31; Putzu et al. [35], RR 0.79, 95% CI 0.63–1.00; and Mohammed et al. [44], RR 0.95, 95% CI 0.89–1.07. Overall, 1 review involving 11 reported studies and 1,043 patients showed reduced mortality, while 3 reviews involving 22 studies and 3,453 patients did not demonstrate a clear effect.

For TPE, most meta-analyses suggested reduced mortality, including Putzu et al. [35], RR 0.63, 95% CI 0.42–0.96; Olive et al. 2023, RR 0.76, 95% CI 0.61–0.94; Zhang et al. [48], RR 0.54, 95% CI 0.33–0.86; Hernandez et al. [51], RR 0.43, 95% CI 0.26–0.72; and Kuklin et al. [52], RR 0.62, 95% CI 0.46–0.83, while Rimmer et al. [26] did not demonstrate a clear effect, RR 0.83, 95% CI 0.45–1.52. Overall, 5 reviews involving 24 reported studies and 872 patients showed favorable findings, while 1 review involving 4 studies and 98 patients did not demonstrate a clear effect.

For CytoSorb, the mortality findings were inconsistent. Steindl et al. [55] suggested reduced mortality, RR 0.64, 95% CI 0.42–0.97, whereas Jiovany et al. [54] and Orban et al. [56] did not demonstrate a clear effect, RR 0.98, 95% CI 0.12–8.25 and RR 0.95, 95% CI 0.58–1.56, respectively. Overall, 1 review involving 9 reported studies and 294 patients showed reduced mortality, while 2 reviews involving 27 studies and 412 patients did not demonstrate a clear effect.

Overall, although several modalities, particularly PMX-HP and TPE, were frequently associated with lower mortality, inconsistency across modalities and imprecision in several meta-analyses indicate that these findings should be interpreted cautiously.

### 3.7. Findings from network meta-analyses

Three eligible network meta-analyses were identified in the updated search and were summarized separately from conventional pairwise meta-analyses because they incorporated both direct and indirect evidence and provided treatment rankings across extracorporeal blood purification modalities [57–59]. Across these NMAs, HA330 and PMX-HP were the modalities most consistently associated with lower mortality compared with standard care. Xing et al. [58] reported significant mortality reductions for HA330 and PMX-HP, with HA330 ranked highest among the evaluated modalities. Chen et al. [57]. found significant mortality reductions for plasma exchange and PMX-HP, with plasma exchange ranked highest. Meco et al. [59]. reported significant mortality reductions for HA330 and PMX-HP, while standard-volume CVVH was associated with increased mortality. However, ranking patterns varied across NMAs, and many comparisons had wide confidence intervals, indicating substantial uncertainty. Therefore, these NMA findings were interpreted as complementary and hypothesis-generating rather than definitive evidence of superiority among modalities. The main findings of the included NMAs are summarized in Table 4.

**Table 4.** Findings from network meta-analyses.

Study	Interventions compared	Relative Effect: RR (95% CI)	Treatment ranking	Interpretation
Xing et al. 2025 [58]	CRRT	0.83 (0.591–1.15)	HA330 > PMX > CRRT > CPFA > OEAHP > CytoSorb	HA330 and PMX-HP showed significant mortality reduction, while other modalities did not show clear benefit.
	CytoSorb	1.83 (0.859–3.97)		
	HA330	0.34 (0.17--0.61)		
	CPFA	0.86 (0.50–1.49)		
	OEAHP	1.16 (0.57–2.35)		
	PMXHP	0.74 (0.57,0.94)		
Chen et al. 2023 [57]	Alteco	0.29 (0.05–1.20)	Plasma exchange > HA330 + High volume CVVH > HA330> oXiris > Standard volume CVVH > CPFA+Standard volume CVVH > High volume CVVH > iHSA > Polymyxin-B > CytoSorb > Very high volume CVVH > Alteco	Plasma exchange and PMX-HP showed significant mortality reduction; most other comparisons were inconclusive.
	CPFA	1.20 (0.82–1.76)		
	CPFA + standard-volume CVVH	0.47 (0.19–1.19)		
	Very high-volume CVVH	0.71 (0.43–1.15)		
	Pulse high-volume CVVH	0.50 (0.05–5.01)		
	High-volume CVVH	0.67 (0.41–1.10)		
	Standard-volume CVVH	0.86 (0.61–1.23)		
	CytoSorb	1.39 (0.97–1.98)		
	HA330	0.61 (0.35–1.09)		
	HA330 + pulse high-volume CVVH	0.63 (0.20–1.91)		
	HA330 + high-volume CVVH	0.58 (0.17–1.93)		
	Immobilized human serum albumin	1.12 (0.54–2.35)		
	oXiris	0.72 (0.29–1.78)		
	Plasma exchange	0.61 (0.42–0.91)		
	Polymyxin-B	0.70 (0.58–0.86)		
	Selective cytopheretic device	1.29 (0.65–1.54)		
Meco et al. 2026 [59]	ALT	0.30 (0.04–1.08)	ALT > HA330> PE > PMX > EFF > CPFA > OXI > IHSA > PHVCVVH > IHD > VHVCVVH > HVCVVH > SVCVVH > CYT	HA330 and PMX-HP showed significant mortality reduction, while standard-volume CVVH was associated with higher mortality in this NMA.
	HA330	0.62 (0.39–0.98)		
	PE	0.73 (0.52–1.01)		
	PMX	0.83 (0.70–0.97)		
	EFF	0.82 (0.51–1.38)		
	CPFA	0.93 (0.72–1.18)		
	OXI	0.90 (0.21–2.85)		
	IHSA	1.10 (0.63–1.89)		
	PHVCVVH	1.16 (0.39–3.40)		
	IHD	1.50 (0.77–2.67)		
	VHVCVVH	1.48 (0.99–2.23)		
	HVCVVH	1.48 (0.97–2.27)		
	SVCVVH	1.50 (1.10–2.09)		
	CYT	1.64 (0.95–2.95)		

### 3.8. Subgroup analysis

**Figure 5.**
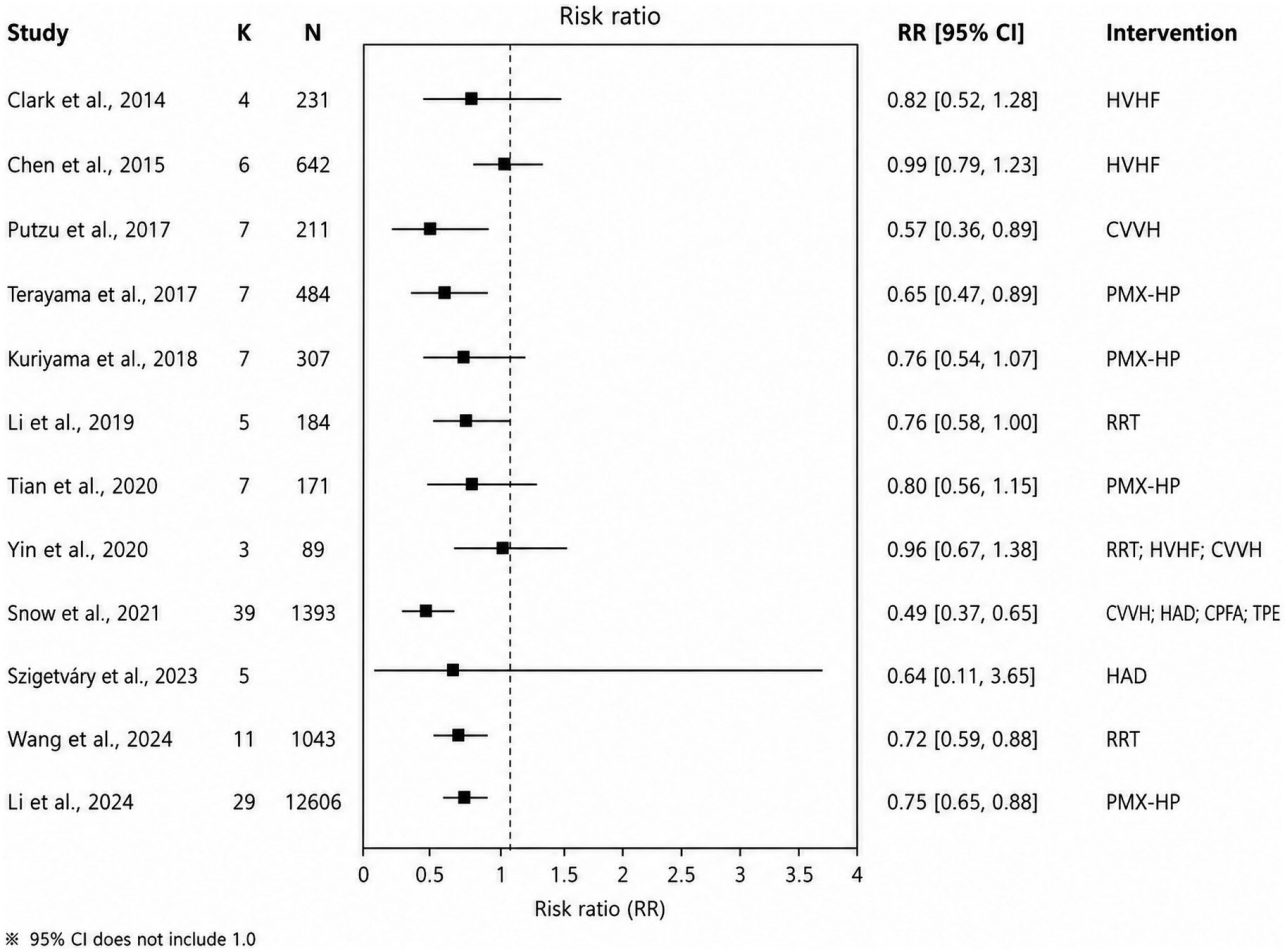
28-day mortality subgroup analysis.

Because mortality was reported using different time horizons across the included reviews, we conducted a descriptive subgroup summary stratified by reported mortality time point, including ICU mortality, in-hospital mortality, 28-day mortality, 60/90-day mortality, and longest follow-up mortality (S3 File). Among these, 28-day mortality was the most frequently reported and relatively comparable time point. In the subgroup of meta-analyses explicitly reporting 28-day mortality (Figure 5), effect estimates generally favored blood purification, with the largest reduction observed in Snow et al. [37] (RR 0.49, 95% CI 0.37–0.65). Statistically significant benefit was also reported by Wang et al. [50] (RR 0.72, 95% CI 0.59–0.88) and Li et al. [53] (RR 0.75, 95% CI 0.65–0.88). At the modality level, the reviews showing significant 28-day mortality benefit mainly involved mixed extracorporeal blood purification strategies including CVVH, HAD, CPFA, and TPE in Snow et al. [37], RRT in Wang et al. [50], and PMX-HP in Li et al. [53]. By contrast, Chen et al. [27], which evaluated HVHF, showed an estimate close to the null (RR 0.99, 95% CI 0.79–1.23).

**Figure 6.**
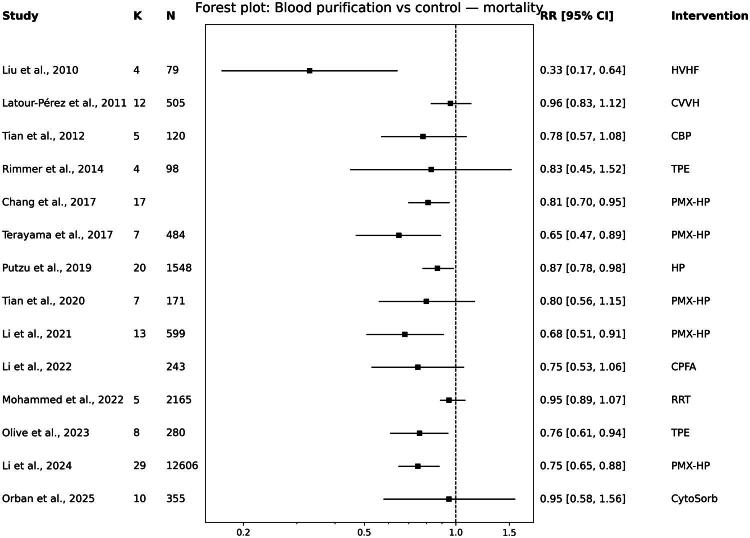
Review-level subgroup summary of meta-analyses focusing on severe sepsis or septic shock.

We further conducted a review-level descriptive subgroup summary of meta-analyses focusing on severe sepsis or septic shock (Figure 6). In this clinically more severe population, effect estimates generally remained in the direction of benefit, but the consistency of findings varied across modalities and study designs. Significant mortality reductions were reported in several reviews evaluating HVHF, PMX-HP, HP, and TPE, including Liu et al. [20], Chang et al. [31], Terayama et al. [32], Putzu et al. [35], Li et al. [41], Olive et al. [45], and Li et al. [53]. However, other reviews focusing on septic shock or severe sepsis showed inconclusive estimates, including Latour-Pérez et al. [21] for CVVH, Tian et al. [23] for CEBP, Rimmer et al. [26] for TPE, Tian et al. [39] for PMX-HP, Li et al. [43] for CPFA, Mohammed et al. [44] for RRT, and Orban et al. [56] for CytoSorb.

**Figure 7.**
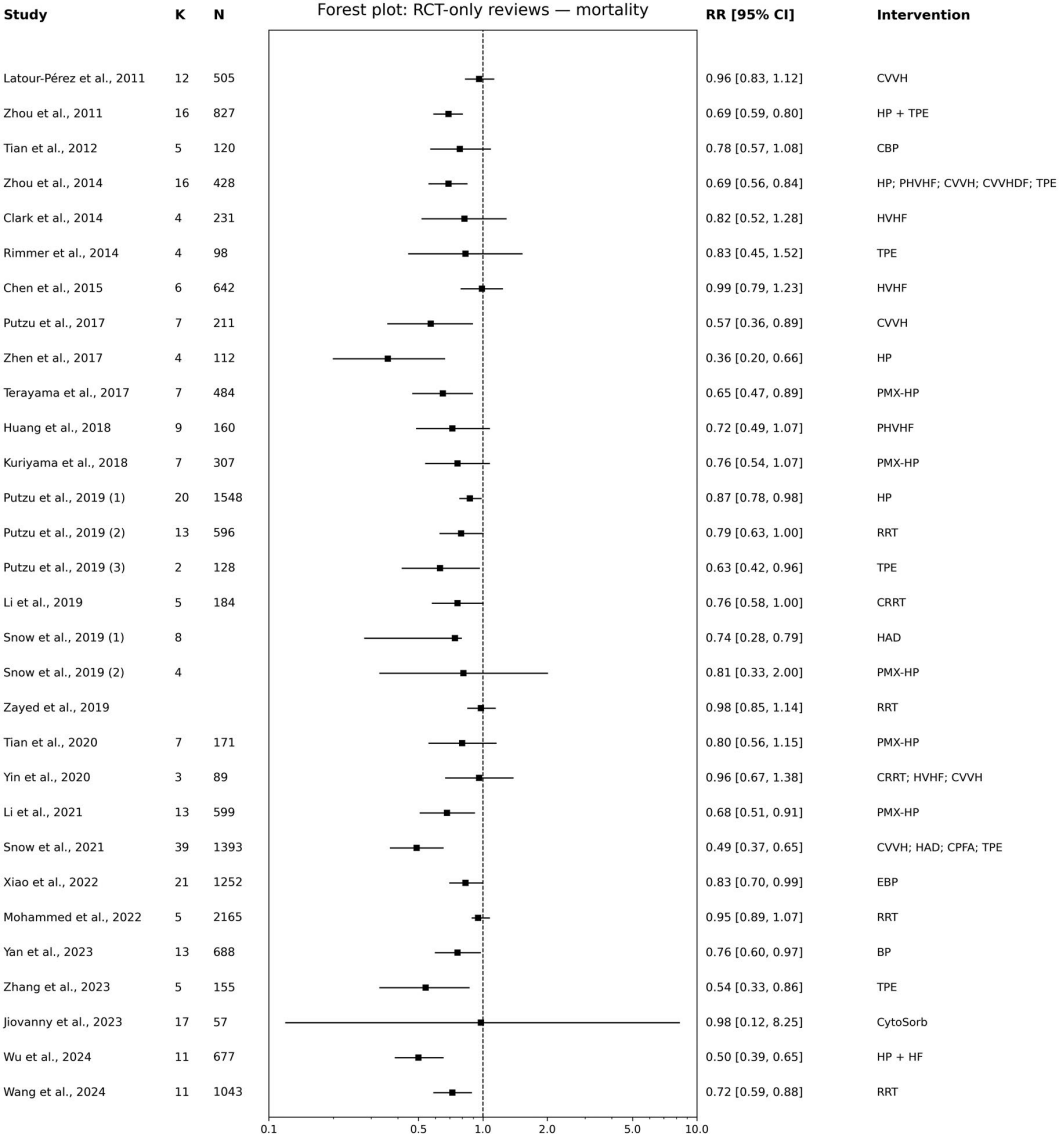
Sensitivity analysis restricted to RCT-only meta-analyses.

We also performed a sensitivity analysis restricted to meta-analyses including randomized controlled trials only (Figure 7). In this analysis, several RCT-only reviews still reported significant mortality reductions, whereas many others showed estimates close to the null or statistically inconclusive findings. For PMX-HP, the RCT-only evidence was inconsistent: Terayama et al. [32] and Li et al. [41] reported significant mortality reductions, while Kuriyama et al. [34], Snow et al. [37], and Tian et al. [39] did not show a clear benefit. These findings suggest that the favorable direction observed in the overall evidence base may be partly influenced by mixed-design reviews, and that modality-specific conclusions, particularly for PMX-HP, should be interpreted cautiously.

### 3.9. Adverse events and safety

Given the substantial heterogeneity in definitions, monitoring approaches, and reporting of adverse events across the included reviews, quantitative synthesis was not feasible; therefore, adverse events were summarized narratively. As shown in Table 5, 7 of the 42 included reviews (16.67%) reported adverse reactions, which were grouped as device-related or patient-related. Device-related adverse events were reported for PMX-HP, including filter clotting/circuit coagulation (categorized as coagulation/circuit), and for hemoperfusion, in which fever was reported (categorized as infection/inflammatory). Patient-related adverse events were most frequently reported for HP—namely allergic reactions (hypersensitivity), hypotension (hemodynamic), and tachycardia (cardiovascular)—and for HVHF, including hypothermia (temperature regulation) and hypophosphatemia (electrolyte/metabolic). Infection was reported in reviews of PMX-HP and pulsed PHVHF and was categorized as infection/inflammatory. Hematologic and bleeding complications were mainly reported for PMX-HP, including anemia (hematologic) and bleeding (hemorrhagic), for PHVHF, including gastrointestinal bleeding (hemorrhagic), and for CytoSorb, including decreased platelet count (hematologic).

**Table 5.** Incidence of adverse reactions.

Type	Blood purification modality	Reported adverse event	Standardized category
Device-related	PMX-HP	Filter clotting / circuit coagulation	Coagulation/circuit
	HP	Fever	Infection/Inflammator
	CytoSorb	Decreased platelet count	Hemorrhagic
Patient-related	HP	Allergic reactions	Hypersensitivity
	HP	Hypotension	Hemodynamic
	HP	Tachycardia	Cardiovascular
	HVHF	Hypothermia	Temperature regulation
	HVHF	Hypophosphatemia	Electrolyte/ metabolic
	PMX-HP, PHVHF	Infection	Infection/ Inflammator
	PMX-HP	Anemia	Hematologic
	PMX-HP	Bleeding	Hemorrhagic
	PHVHF	Gastrointestinal bleeding	Hemorrhagic

## 4. Discussion

We included 42 systematic reviews and meta-analyses published between 2007 and 2026, covering a heterogeneous set of EEBP modalities, including PMX-HP, HP, HVHF, CBP/EBP, RRT, TPE, CPFA, and CytoSorb. Across the included evidence, mortality was the most consistently reported clinical endpoint. Overall, most meta-analyses reported point estimates favoring EBP versus conventional care, although the magnitude of benefit varied across modalities and reviews and statistical significance was not uniform. In qualitative terms, PMX-HP showed the most consistent review-level direction toward lower mortality, whereas estimates for RRT-based strategies appeared closest to the null and least consistently favorable across reviews. However, these modality-specific patterns should be interpreted cautiously because the overall certainty of evidence for mortality was low, and the apparent consistency of PMX-HP should not be considered definitive evidence of survival benefit. Beyond mortality, evidence was frequently directed toward physiological severity indices, hemodynamic variables, and inflammatory biomarkers. These outcomes may partly reflect not only mediator removal but also broader immunomodulatory effects, including changes in leukocyte activation, monocyte/macrophage and neutrophil responses, and endothelial–immune interactions [7,60]. By contrast, safety outcomes were reported in only a small proportion of reviews and could not be quantitatively synthesized due to inconsistent definitions and reporting. Taken together, this umbrella review delineates the current evidence landscape for EBP in sepsis, while underscoring that any apparent differences between modalities should be interpreted cautiously given substantial heterogeneity in patient populations, intervention protocols and timing, comparator care, and potential overlap of primary studies across reviews.

A key feature of the present umbrella review is the modality-specific synthesis of extracorporeal blood purification strategies, which allows more clinically meaningful comparisons across fundamentally different technologies. Although these interventions are often grouped under the broad term of blood purification, they differ substantially in therapeutic targets and clearance mechanisms [46,54,55]. PMX-HP is designed primarily for endotoxin adsorption; hemoperfusion and hemoadsorption remove a broader spectrum of inflammatory mediators depending on cartridge and membrane properties; hemofiltration-based strategies such as HVHF or PHVHF aim to enhance convective clearance; TPE exchanges plasma to remove circulating mediators and potentially replenish protective plasma components; and CPFA integrates plasma separation with adsorption [61–68]. These mechanistic differences suggest that combining all extracorporeal modalities into a single pooled estimate may obscure clinically meaningful variation. Therefore, presenting evidence by modality is essential for interpreting the current evidence base.

The modality-specific findings should be interpreted within the temporal evolution of sepsis blood purification research. The included evidence spans nearly two decades, during which the rationale and clinical positioning of extracorporeal blood purification changed substantially. Early evidence was largely shaped by high-volume hemofiltration and continuous hemofiltration, based on the assumption that enhanced convective clearance could reduce circulating inflammatory mediators in severe sepsis and septic shock [69]. During the 2010s, research expanded toward more complex or targeted strategies, including coupled plasma filtration adsorption, hemoperfusion, plasma exchange, and endotoxin-directed adsorption [43]. More recently, renewed interest in adsorptive technologies and network meta-analytic comparisons has shifted the field from evaluating blood purification as a uniform adjunctive therapy toward comparing modality-specific effects in selected patient groups. These temporal changes may confound cross-modality comparisons because reviews from different periods differed not only in the devices evaluated, but also in sepsis definitions, patient selection, timing of initiation, treatment intensity, co-interventions, and comparator care [70]. In parallel, the transition from SIRS-based/Sepsis-2 concepts to Sepsis-3, together with evolving standards such as earlier antimicrobial therapy, timely source control, vasopressor optimization, and more conservative fluid strategies, may have changed baseline mortality risk and modified the marginal effect of adjunctive blood purification [1,71].

The interpretation of PMX-HP requires particular caution within this temporal context. Although several earlier meta-analyses and some Asian-population syntheses reported a favorable mortality signal, this evidence must be weighed against the EUPHRATES trial, the largest randomized trial of PMX-HP in endotoxemic septic shock, which did not show a significant 28-day mortality benefit in the overall study population [72–74]. This creates an important tension between the relatively consistent favorable direction observed in some review-level syntheses and the neutral result of the largest and more rigorous trial. Although post-hoc findings from EUPHRATES suggested that patients with a more clearly defined endotoxin-related phenotype might derive greater benefit, these findings should be interpreted as hypothesis-generating and support a phenotype-sensitive interpretation rather than routine use of PMX-HP in unselected patients with sepsis or septic shock. Moreover, trial sequential analysis has suggested that the apparent survival benefit of PMX-HP may have been overestimated by early, underpowered trials and repeated meta-analyses [67]. Therefore, in this umbrella review, PMX-HP should be understood as showing a relatively consistent review-level signal toward benefit, but not as definitive evidence of mortality reduction.

A major challenge in interpreting the mortality evidence for extracorporeal blood purification is that ‘mortality’ is reported using multiple, nonequivalent time horizons (ICU, in-hospital, 28/30-day, 60/90-day, longest follow-up, or all-cause), and the choice of endpoint can materially change the apparent signal. For judging whether blood purification improves outcomes in sepsis in a biologically and clinically meaningful way, 28-day (or 30-day) all-cause mortality is typically decision-relevant and comparable endpoint because it aligns with the hypothesized treatment window—early modulation of dysregulated host responses during shock and evolving organ dysfunction—and it is less distorted by post-ICU discharge practices than ICU or in-hospital mortality [75]. In contrast, ICU/in-hospital mortality is highly sensitive to institutional discharge/transfer policies and bed availability, which can create between-study differences unrelated to treatment effect, whereas 60/90-day or longest follow-up mortality increasingly captures late deaths driven by downstream complications, rehabilitation trajectories, and competing non-sepsis risks, potentially diluting an early treatment effect and inflating heterogeneity across studies. Therefore, presenting a subgroup restricted to explicitly defined 28-day mortality improves interpretability by standardizing the time horizon, but residual variability should still be expected because endpoint harmonization does not remove heterogeneity in case-mix, timing of initiation, treatment intensity/duration, and co-interventions; future trials and meta-analyses would be strengthened by prespecifying 28/30-day all-cause mortality as the primary endpoint with 90-day mortality as a key secondary outcome to assess durability.

The variability in mortality findings across reviews was further supported by the subgroup and sensitivity analyses, but these analyses also highlight the gap between biologic plausibility and phenotype-stratified clinical evidence. In the review-level subgroup summary of meta-analyses focusing on severe sepsis or septic shock, effect estimates generally favored blood purification, but findings remained inconsistent across modalities and study designs. Some reviews of HVHF, PMX-HP, HP, and TPE reported significant mortality reductions, whereas reviews of CVVH, CBP, CPFA, RRT, CytoSorb, and some PMX-HP analyses showed inconclusive results [76–78]. This suggests that greater clinical severity alone may not be sufficient to identify a consistently responsive population for extracorporeal blood purification.

Although post-hoc trial evidence and biologic plausibility suggest that endotoxin-adsorbing strategies such as PMX-HP may be more relevant in endotoxin-mediated shock, and that adsorptive approaches may be more suitable for hyperinflammatory states, the included reviews did not provide direct review-level evidence that mortality effects differ across predefined sepsis phenotypes [79–81]. Therefore, phenotype-directed EBP selection should be interpreted as a hypothesis-generating framework rather than an established evidence-based strategy. The RCT-only sensitivity analysis also tempered the overall interpretation: after excluding mixed-design reviews, mortality effects were less uniform, particularly for PMX-HP, for which some RCT-only syntheses reported benefit whereas others did not show a clear reduction. These findings indicate that favorable signals in the overall evidence base may partly reflect differences in study design composition and the influence of non-randomized evidence. Observational studies in sepsis blood purification are especially vulnerable to indication bias and residual confounding, because extracorporeal therapies may be selectively applied to patients with more severe shock, AKI, fluid overload, or refractory inflammation, or concentrated in experienced centers. Future trials and living systematic reviews should move beyond broad severity categories and prospectively evaluate predefined enrichment factors such as endotoxin activity, infection source, inflammatory or immune phenotype, AKI status, and timing of intervention [82].

Moreover, many reviews grouped clinically diverse sepsis phenotypes and heterogeneous treatment parameters under broad modality labels, limiting comparability and increasing the risk that observed differences between techniques reflect differences in populations and clinical context [12]. These issues collectively indicate that future evidence synthesis would benefit from stricter modality definitions, prespecified effect modifiers aligned with sepsis biology and clinical decision points, and more transparent propagation of bias considerations into conclusions [78].

Several limitations should also be acknowledged. First, substantial clinical heterogeneity in sepsis phenotypes, intervention timing, treatment intensity and duration, co-interventions, and comparator care complicates comparisons between modalities. Second, although the corrected covered area indicated only slight overlap of primary studies across reviews, overlap should not be ignored completely. Some influential trials may still recur within specific modality-focused evidence clusters, making certain modality-specific signals, such as those for PMX-HP, appear more consistent than they would in a fully independent evidence base. Third, safety outcomes were infrequently and inconsistently reported, precluding quantitative synthesis; this should be regarded as a major limitation for clinical decision-making. Future systematic reviews should standardize the definitions and reporting of adverse events, for example by using established criteria such as the Common Terminology Criteria for Adverse Events (CTCAE) [83]. In addition, because only English- and Chinese-language records were included, potential language bias cannot be excluded.

## 5. Conclusion

In conclusion, this umbrella review synthesizes review-level evidence on EBP in sepsis. Mortality estimates generally favored EBP over conventional care; PMX-HP showed a more consistently favorable direction of effect, whereas RRT-based strategies were generally closest to no effect, and other modalities yielded more variable estimates. Interpretation is constrained by substantial clinical and methodological heterogeneity, overlap of primary studies across reviews, and the predominantly low methodological quality of the included evidence base. Future work should prioritize adequately powered trials with standardized intervention protocols and prespecified, clinically relevant effect modifiers, alongside higher-fidelity evidence syntheses that can support modality- and phenotype-informed decision making.

## Supplementary Material

S7 Article deleted after reading the full text.docx

S3 Mortality effect estimates stratified by timepoint.pdf

S5 File GRADE.pdf

S4 File Study overlap CCA.pdf

S2 File Search strategies of eight databases.pdf

S6 Effects of blood purification modalities on mortality.docx

S1 File PRISMA 2020 Checklist for Umbrella Review.docx

## Data Availability

All data analyzed in this study are derived from previously published studies, which are cited within the article. No new data were generated. The extracted and synthesized data that support the findings of this study are available from the corresponding author upon reasonable request.

## References

[1] Singer M, Deutschman CS, Seymour CW, et al. The third international consensus definitions for sepsis and septic shock (sepsis‐3). JAMA. 2016;315(8):801–810. doi: 10.1001/jama.2016.0287. 26903338 PMC4968574

[2] Rudd KE, Johnson SC, Agesa KM, et al. Global, regional, and national sepsis incidence and mortality, 1990–2017: analysis for the Global Burden of Disease Study. Lancet. 2020;395(10219):200–211. doi: 10.1016/S0140-6736(19)32989-7. 31954465 PMC6970225

[3] Fleischmann C, Scherag A, Adhikari NK, et al. Assessment of global incidence and mortality of hospital-treated sepsis. Current estimates and limitations. Am J Respir Crit Care Med. 2016;193(3):259–272. doi: 10.1164/rccm.201504-0781OC. 26414292

[4] Prescott HC, Angus DC. Enhancing recovery from sepsis: a review. JAMA. 2018;319(1):62–75. doi: 10.1001/jama.2017.17687. 29297082 PMC5839473

[5] Fan JB, Li QY, Feng XF, et al. The “cytokine storm” in infection and sepsis: win the battle but lose the war. Mil Med Res. 2026;12(1):95. doi: 10.1186/s40779-025-00678-0. 41521316 PMC12794442

[6] Berlot G, Tomasini A, Zanchi S, et al. The techniques of blood purification in the treatment of sepsis and other hyperinflammatory conditions. J Clin Med. 2023;12(5):1723. doi: 10.3390/jcm12051723. 36902510 PMC10002609

[7] Jarczak D, Kluge S, Nierhaus A. Septic hyperinflammation – is there a role for extracorporeal blood purification techniques? Int J Mol Sci. 2024;25(6):3120. doi: 10.3390/ijms25063120. 38542094 PMC10970398

[8] Sun Y, Huang X, Hong B, et al. The blood purification therapy-based strategy effectively rescued the severe falciparum malaria patient experiencing cytokine storm-driven MODS. Front Cell Infect Microbiol. 2026;15:1682892. doi: 10.3389/fcimb.2025.1682892. 41552719 PMC12808470

[9] Atan R, Crosbie DC, Bellomo R. Techniques of extracorporeal cytokine removal: a systematic review of human studies. Ren Fail. 2013;35(8):1061–1070. doi: 10.3109/0886022X.2013.815089. 23866032

[10] Gaudry S, Hajage D, Schortgen F, et al. Initiation strategies for renal-replacement therapy in the intensive care unit. N Engl J Med. 2016;375(2):122–133. doi: 10.1056/NEJMoa1603017. 27181456

[11] Payen D, Mateo J, Cavaillon JM, et al. Impact of continuous venovenous haemofiltration on organ failure during the early phase of severe sepsis: a randomized controlled trial. Crit Care Med. 2009;37(3):803–810. doi: 10.1097/CCM.0b013e3181962316. 19237881

[12] Bottari G, Ranieri VM, Ince C, et al. Use of extracorporeal blood purification therapies in sepsis: the current paradigm, available evidence, and future perspectives. Crit Care. 2024;28(1):432. doi: 10.1186/s13054-024-05220-7. 39722012 PMC11670469

[13] Mulrow C, Langhorne P, Grimshaw J. Integrating heterogeneous pieces of evidence in systematic reviews. Ann Intern Med. 1997;127(11):989–995. doi: 10.7326/0003-4819-127-11-199712010-00008. 9412305

[14] Gates M, Gates A, Pieper D, et al. Reporting guideline for overviews of reviews of healthcare interventions: development of the PRIOR statement. BMJ. 2022;378:e070849. doi: 10.1136/bmj-2022-070849. 35944924 PMC9361065

[15] Papatheodorou SI, Evangelou E. Umbrella reviews: what they are and why we need them. Methods Mol Biol. 2022;2345:135–146. doi: 10.1007/978-1-0716-1566-9_8. 34550588

[16] Lunny C, Brennan SE, McDonald S, et al. Toward a comprehensive evidence map of overview of systematic review methods: paper 2-risk of bias assessment; synthesis, presentation and summary of the findings; and assessment of the certainty of the evidence. Syst Rev. 2018;7(1):159. doi: 10.1186/s13643-018-0784-8. 30314530 PMC6186052

[17] López-López JA, Rubio-Aparicio M, Sánchez-Meca J. Overviews of reviews: concept and development. Psicothema. 2022;34(2):175–181. doi: 10.7334/psicothema2021.586. 35485529

[18] Liberati A, Altman DG, Tetzlaff J, et al. The PRISMA statement for reporting systematic reviews and meta-analyses of studies that evaluate healthcare interventions: explanation and elaboration. BMJ. 2009;339:b2700. doi: 10.1136/bmj.b2700. 19622552 PMC2714672

[19] Cruz DN, Perazella MA, Bellomo R, et al. Effectiveness of polymyxin B-immobilized fiber column in sepsis: a systematic review. Crit Care. 2007;11(2):R47. doi: 10.1186/cc5780. 17448226 PMC2206475

[20] Liu Y, Xie J, Guo F, et al. Effects of high volume hemofiltration on mortality in patients with septic shock: a meta-analysis. Zhonghua Yi Xue Za Zhi. 2010;90(37):2601–2606. doi: 10.3760/cma.j.issn.0376-2491.2010.37.003. 21162924

[21] Latour-Pérez J, Palencia-Herrejón E, Gómez-Tello V, et al. Intensity of continuous renal replacement therapies in patients with severe sepsis and septic shock: a systematic review and meta-analysis. Anaesth Intensive Care. 2011;39(3):373–383. doi: 10.1177/0310057X1103900307. 21675056

[22] Zhou F, Peng Z, Murugan R, et al. Does blood purification improve survival in sepsis? A meta-analysis of published trials. Crit Care Med. 2011;39(12):209. doi: 10.1097/01.ccm.0000408627.24229.88.21178542

[23] Hongliang T, Rong Z, Xiaojing W, et al. The effects of continuous blood purification for SIRS/MODS patients: a systematic review and meta-analysis of randomized controlled trials. ISRN Hematol. 2012;2012:986795–986710. doi: 10.5402/2012/986795. 23056956 PMC3463946

[24] Zhou F, Peng Z, Murugan R, et al. Blood purification and mortality in sepsis: a meta-analysis of randomized trials. Crit Care Med. 2013;41(9):2209–2220. doi: 10.1097/CCM.0b013e31828cf412. 23860248 PMC3758418

[25] Clark E, Molnar AO, Joannes-Boyau O, et al. High-volume hemofiltration for septic acute kidney injury: a systematic review and meta-analysis. Crit Care. 2014;18(1):R7. doi: 10.1186/cc13184. 24398168 PMC4057068

[26] Rimmer E, Houston BL, Kumar A, et al. The efficacy and safety of plasma exchange in patients with sepsis and septic shock: a systematic review and meta-analysis. Crit Care. 2014;18(6):699. doi: 10.1186/s13054-014-0699-2. 25527094 PMC4318234

[27] Chen Y, Fang C, Cha J, et al. Effects of high-volume hemofiltration for septic acute kidney failure: a meta-analysis. Anhui Med J. 2015;36(03):278–281.

[28] Gong X, Wang R, Li G. Effect of timing of initiation of renal replacement therapy on prognosis in septic patients with acute kidney injury: a meta analysis. Zhonghua Wei Zhong Bing Ji Jiu Yi Xue. 2015;27(9):712–717. 26955695

[29] Putzu A, Fang MX, Boscolo Berto M, et al. Blood purification with continuous veno-venous hemofiltration in patients with sepsis or ARDS: a systematic review and meta-analysis. Minerva Anestesiol. 2017;83(8):867–877. doi: 10.23736/S0375-9393.17.11946-2. 28607338

[30] Zhen J, Li L, Yan J. Systematic review on the clinical effect of hemoperfusion with HA330 resin cartridge on treatment of sepsis. J Pract Shock. 2017;1(02):95–100.

[31] Chang T, Tu YK, Lee CT, et al. Effects of polymyxin B hemoperfusion on mortality in patients with severe sepsis and septic shock: a systemic review, meta-analysis update, and disease severity subgroup meta-analysis. Crit Care Med. 2017;45(8):e858–e864. doi: 10.1097/CCM.0000000000002362. 28445237 PMC5515642

[32] Terayama T, Yamakawa K, Umemura Y, et al. Polymyxin B hemoperfusion for sepsis and septic shock: a systematic review and meta-analysis. Surg Infect (Larchmt). 2017;18(3):225–233. doi: 10.1089/sur.2016.168. 28092497

[33] Huang H, Liu J, Chen X, et al. Therapeutic effect of pulse high volume hemofiltration for treatment of patients with sepsis: a systemic review and Meta-analysis. Chin J Integr Tradit West Med Intensive Crit Care. 2018;25(2):113–119. doi: 10.3969/j.issn.1008-9691.2018.02.001.

[34] Kuriyama A, Katsura M, Urushidani S, et al. Impact of polymyxin B hemoperfusion in the treatment of patients with sepsis and septic shock: a meta-analysis of randomized controlled trials. Ann Transl Med. 2018;6(11):206–206. doi: 10.21037/atm.2018.05.41. 30023369 PMC6035977

[35] Putzu A, Schorer R, Lopez-Delgado JC, et al. Blood purification and mortality in sepsis and septic shock: a systematic review and meta-analysis of randomized trials. Anesthesiology. 2019;131(3):580–593. doi: 10.1097/ALN.0000000000002820. 31246600

[36] Li Y, Li H, Zhang D. Timing of continuous renal replacement therapy in patients with septic AKI: a systematic review and meta-analysis. Medicine (Baltimore). 2019;98(33):e16800. doi: 10.1097/MD.0000000000016800. 31415389 PMC6831327

[37] Snow TAC, Littlewood S, Corredor C, et al. Effect of extracorporeal blood purification on mortality in sepsis: a meta-analysis and trial sequential analysis. Blood Purif. 2021;50(4-5):462–472. doi: 10.1159/000510982. 33113533

[38] Zayed Y, Kheiri B, Rashdan L, et al. Early versus late initiation of renal replacement therapy in patients with sepsis: a meta-analysis of randomized controlled trials. Am Thorac Soc Int Conf. 2019;17(22):A6501. doi: 10.1164/ajrccm-conference.2019.199.1_MeetingAbstracts.A6501.

[39] Tian X, Chen Z, He H, et al. The effect of polymyxin B hemoperfusion on prognosis of patients with sepsis and septic shock: a meta-analysis. Chin J Respir Crit Care Med. 2020;19(01):16–21.

[40] Yin F, Zhang F, Liu S, et al. The therapeutic effect of high-volume hemofiltration on sepsis: a systematic review and meta-analysis. Ann Transl Med. 2020;8(7):488–488. doi: 10.21037/atm.2020.03.48. 32395532 PMC7210131

[41] Li X, Liu C, Mao Z, et al. Effectiveness of polymyxin B-immobilized hemoperfusion against sepsis and septic shock: a systematic review and meta-analysis. J Crit Care. 2021;63:187–195. doi: 10.1016/j.jcrc.2020.09.007. 33012579

[42] Liuniu X, Yanxia Z, Shusheng L. Effect of extracorporeal hemopurification for clinical prognosis and cytokine levels of septic: a meta-analysis. Clin Focus. 2022;37(1):5–13.

[43] Li Y, Li H, Guo J, et al. Coupled plasma filtration adsorption for the treatment of sepsis or septic shock: a systematic review and meta-analysis. BMC Infect Dis. 2022;22(1):714. doi: 10.1186/s12879-022-07689-5. 36038815 PMC9422100

[44] Somaili M. Early versus delayed strategies for renal replacement therapy initiation in adult patients with severe acute kidney injury complicating septic shock: a systematic review and meta-analysis. Saudi J Kidney Dis Transpl. 2022;33(3):449–486. doi: 10.4103/1319-2442.385969. 37843147

[45] Lee OPE, Kanesan N, Leow EH, et al. Survival benefits of therapeutic plasma exchange in severe sepsis and septic shock: a systematic review and meta-analysis. J Intensive Care Med. 2023;38(7):598–611. doi: 10.1177/08850666231170775. 37097910

[46] Yan J, Zhang Y, Zhang J. Clinical efficacy of blood purification in the treatment of sepsis: a meta-analysis of the last 5 years. Clin Lab. 2023;69(6):10. doi: 10.7754/Clin.Lab.2022.220931. 37307130

[47] Szigetváry CE, Turan C, Kovács EH, et al. Hemoadsorption as adjuvant therapy in acute respiratory distress syndrome (ARDS): A systematic review and meta-analysis. Biomedicines. 2023;11(11):3068. doi: 10.3390/biomedicines11113068. 38002070 PMC10669540

[48] Zhang L, Zhao XY, Guo SY, et al. An inquiry into the treatment of sepsis using plasma exchange therapy: A systematic review and meta-analysis. Int Wound J. 2023;20(6):1979–1986. doi: 10.1111/iwj.14059. 36717980 PMC10332988

[49] Wu L, Wang L, Luo L, et al. Meta analysis of the effects of hemoperfusion combined with hemofiltration on inflammatory mediatorsand prognosis in sepsis. Chin J Blood Purif. 2024;23(05):338–341.

[50] Wang Q, Liu F, Tao W, et al. Timing of renal replacement therapy in patients with sepsis-associated acute kidney injury: A systematic review and meta-analysis. Aust Crit Care. 2024;37(2):369–379. doi: 10.1016/j.aucc.2023.06.011. 37734999

[51] Hernandez GN, Francis AJ, Hamid P. Enhancing survival in septic shock: a systematic review and meta-analysis of the efficacy of plasma exchange therapy. Cureus. 2024;16(5):e60947. doi: 10.7759/cureus.60947. 38910774 PMC11193551

[52] Kuklin V, Sovershaev M, Bjerner J, et al. Influence of therapeutic plasma exchange treatment on short-term mortality of critically ill adult patients with sepsis-induced organ dysfunction: a systematic review and meta-analysis. Crit Care. 2024;28(1):12. doi: 10.1186/s13054-023-04795-x. 38178170 PMC10768220

[53] Li C, Zhang J, Yang P, et al. The role of polymyxin B-immobilized hemoperfusion in reducing mortality and enhancing hemodynamics in patients with sepsis and septic shock: A systematic review and meta-analysis. Heliyon. 2024;10(13):e33735. doi: 10.1016/j.heliyon.2024.e33735. 39040355 PMC11261863

[54] Saldaña-Gastulo JJC, Llamas-Barbarán MDR, Coronel-Chucos LG, et al. Cytokine hemoadsorption with CytoSorb^®^ in patients with sepsis: a systematic review and meta-analysis. Crit Care Sci. 2023;35(2):217–225. doi: 10.5935/2965-2774.20230289-en. 37712812 PMC10406402

[55] Steindl D, Schroeder T, Krannich A, et al. Hemoadsorption in the management of septic shock: a systematic review and meta-analysis. J Clin Med. 2025;14(7):2285. doi: 10.3390/jcm14072285. 40217734 PMC11989519

[56] Orban C, Bratu A, Agapie M, et al. To hemoadsorb or not to hemoadsorb-do we have the answer yet? An updated meta-analysis on the use of cytosorb in sepsis and septic shock. Biomedicines. 2025;13(7):180. doi: 10.3390/biomedicines13010180. 39857764 PMC11762373

[57] Chen JJ, Lai PC, Lee TH, et al. Blood purification for adult patients with severe infection or sepsis/septic shock: a network meta-analysis of randomized controlled trials. Crit Care Med. 2023;51(12):1777–1789. doi: 10.1097/CCM.0000000000005991. 37470680 PMC10645104

[58] Xing H, Wei Y, Zhang D, et al. Comparing adsorptive blood purification modalities for sepsis patients: A systematic review and network meta-analysis. Respir Med. 2025;239:107994. doi: 10.1016/j.rmed.2025.107994. 39952412

[59] Meco M, Agosteo E, Zulli P, et al. Effects on mortality of blood purification techniques in severe septic shock patients. An updated Bayesian network meta-analysis of randomized controlled trials. J Crit Care. 2026;92:155330. doi: 10.1016/j.jcrc.2025.155330. 41237653

[60] Rimmelé T, Kaynar AM, McLaughlin JN, et al. Leukocyte capture and modulation of cell-mediated immunity during human sepsis: an ex vivo study. Crit Care. 2013;17(2):R59. doi: 10.1186/cc12587. 23531333 PMC3672497

[61] Rizo-Topete L, Molano-Triviño A, Vega-Vega O, et al. Extracorporeal blood purification therapies in latin america – bridging gaps in availability and training. Blood Purif. 2026;55(5):274–286. doi: 10.1159/000550109. 41511903

[62] Yap HJ, Chen YC, Fang JT, et al. Combination of continuous renal replacement therapies (CRRT) and extracorporeal membrane oxygenation (ECMO) for advanced cardiac patients. Ren Fail. 2003;25(2):183–193. doi: 10.1081/jdi-120018719. 12739825

[63] Martins Costa A, Halfwerk F, Wiegmann B, et al. Trends, advantages and disadvantages in combined extracorporeal lung and kidney support from a technical point of view. Front Med Technol. 2022;4:909990. doi: 10.3389/fmedt.2022.909990. 35800469 PMC9255675

[64] Cho Y, Bello AK, Levin A, et al. Peritoneal dialysis use and practice patterns: an international survey study. Am J Kidney Dis. 2021;77(3):315–325. doi: 10.1053/j.ajkd.2020.05.032. 32800844

[65] Bello AK, Levin A, Lunney M, et al. Status of care for end stage kidney disease in countries and regions worldwide: international cross sectional survey. BMJ. 2019;367:l5873. doi: 10.1136/bmj.l5873. 31672760

[66] Chang WH, Hu TY, Kuo LK. Sequential versus non-sequential polymyxin b hemoperfusion in severe sepsis and septic shock: a real-world cohort analysis of survival in an Asian ICU. Diagnostics (Basel). 2026;16(1):173. doi: 10.3390/diagnostics16010173. 41515668 PMC12785834

[67] Fujii T, Ganeko R, Kataoka Y, et al. Polymyxin B-immobilized hemoperfusion and mortality in critically ill adult patients with sepsis/septic shock: a systematic review with meta-analysis and trial sequential analysis. Intensive Care Med. 2018;44(2):167–178. doi: 10.1007/s00134-017-5004-9. 29204670

[68] Borthwick EM, Hill CJ, Rabindranath KS, et al. High-volume haemofiltration for sepsis. Cochrane Database Syst Rev. 2013;1:CD008075. doi: 10.1002/14651858.CD008075.pub2. 23440825

[69] Venkataraman R, Subramanian S, Kellum JA. Clinical review: extracorporeal blood purification in severe sepsis. Crit Care. 2003;7(2):139–145. doi: 10.1186/cc1889. 12720560 PMC270630

[70] Evans L, Rhodes A, Alhazzani W, et al. Surviving sepsis campaign: international guidelines for management of sepsis and septic shock 2021. Intensive Care Med. 2021;47(11):1181–1247. doi: 10.1007/s00134-021-06506-y. 34599691 PMC8486643

[71] Protti A, Fortunato F, Artoni A, et al. Platelet mitochondrial dysfunction in critically ill patients: comparison between sepsis and cardiogenic shock. Crit Care. 2015;19(1):39. doi: 10.1186/s13054-015-0762-7. 25757508 PMC4338849

[72] Chen SH, Chan WS, Liu CM, et al. Effects of endotoxin adsorber hemoperfusion on sublingual microcirculation in patients with septic shock: a randomized controlled trial. Ann Intensive Care. 2020;10(1):80. doi: 10.1186/s13613-020-00699-z. 32533380 PMC7290141

[73] Osawa I, Goto T, Kudo D, et al. Targeted therapy using polymyxin B hemadsorption in patients with sepsis: a post-hoc analysis of the JSEPTIC-DIC study and the EUPHRATES trial. Crit Care. 2023;27(1):245. doi: 10.1186/s13054-023-04533-3. 37344804 PMC10286480

[74] Dellinger RP, Bagshaw SM, Antonelli M, et al. Effect of targeted polymyxin B hemoperfusion on 28-day mortality in patients with septic shock and elevated endotoxin level: the EUPHRATES randomized clinical trial. JAMA. 2018;320(14):1455–1463. doi: 10.1001/jama.2018.14618. 30304428 PMC6233793

[75] Peng J, Li L, Wang G, et al. Association of 41 circulating inflammatory factors, C-reactive protein, and procalcitonin with sepsis risk and 28-day mortality: A bidirectional Mendelian randomization and mediation analysis. Adv Clin Exp Med. 2026;35(1):45–55. doi: 10.17219/acem/203155. 41489866

[76] Wang W, Liu CF. Sepsis heterogeneity. World J Pediatr. 2023;19(10):919–927. doi: 10.1007/s12519-023-00689-8. 36735197

[77] Marshall JC. Why have clinical trials in sepsis failed? Trends Mol Med. 2014;20(4):195–203. doi: 10.1016/j.molmed.2014.01.007. 24581450

[78] Antcliffe DB, Burrell A, Boyle AJ, et al. Sepsis subphenotypes, theragnostics and personalized sepsis care. Intensive Care Med. 2025;51(4):756–768. doi: 10.1007/s00134-025-07873-6. 40163135 PMC12055953

[79] Shiga H, Hirasawa H, Nishida O, et al. Continuous hemodiafiltration with a cytokine-adsorbing hemofilter in patients with septic shock: a preliminary report. Blood Purif. 2014;38(3-4):211–218. doi: 10.1159/000369377. 25531978

[80] Reddy K, Sinha P, O’Kane CM, et al. Subphenotypes in critical care: translation into clinical practice. Lancet Respir Med. 2020;8(6):631–643. doi: 10.1016/S2213-2600(20)30124-7. 32526190

[81] Gordon AC, Alipanah-Lechner N, Bos LD, et al. From ICU syndromes to ICU subphenotypes: consensus report and recommendations for developing precision medicine in the ICU. Am J Respir Crit Care Med. 2024;210(2):155–166. doi: 10.1164/rccm.202311-2086SO. 38687499 PMC11273306

[82] Forero DA, Abreu SE, Tovar BE, et al. Automated analyses of risk of bias and critical appraisal of systematic reviews (ROBIS and AMSTAR 2): a comparison of the performance of 4 large language models. J Am Med Inform Assoc. 2025;32(9):1471–1476. doi: 10.1093/jamia/ocaf117. 40680299 PMC12361857

[83] Basch E, Reeve BB, Mitchell SA, et al. Development of the National Cancer Institute’s patient-reported outcomes version of the common terminology criteria for adverse events (PRO-CTCAE). J Natl Cancer Inst. 2014;106(9):dju244. doi: 10.1093/jnci/dju244. 25265940 PMC4200059

