# Supplementary material for: Effects on mortality of different blood purification techniques in sepsis patients: an umbrella review of systematic reviews and meta-analyses
Source: Ren Fail. 2026 Jul 16;48(1):2698155. doi: 10.1080/0886022X.2026.2698155 (PMC13378714; doi:10.1080/0886022X.2026.2698155)
Supplement: S7 Article deleted after reading the full text.docx [file IRNF_A_2698155_SM5763.docx]

| **Study** | **Exclude the cause** |
| --- | --- |
| 连续性血液净化治疗严重烧伤脓毒症临床疗效的Meta分析 | Irrelevant outcomes |
| Effectiveness of polymyxin B-immobilized fiber column in sepsis: a systematic review | Irrelevant outcomes |
| Early versus Delayed Strategies for Renal Replacement Therapy Initiation in Adult Patients with Severe Acute Kidney Injury Complicating Septic Shock: A Systematic Review and Meta-analysis | Irrelevant outcomes |
| Survival Benefits of Therapeutic Plasma Exchange in Severe Sepsis and Septic Shock: A Systematic Review and Meta-analysis | Irrelevant outcomes |
| Resuscitation Fluids in Septic Shock: A Network Meta-Analysis of Randomized Controlled Trials | Irrelevant intervention |
| Fluid resuscitation management in patients with sepsis and septic shock: a network meta-analysis | Irrelevant intervention |
