# Supplementary material for: Effects on mortality of different blood purification techniques in sepsis patients: an umbrella review of systematic reviews and meta-analyses
Source: Ren Fail. 2026 Jul 16;48(1):2698155. doi: 10.1080/0886022X.2026.2698155 (PMC13378714; doi:10.1080/0886022X.2026.2698155)
Supplement: S3 Mortality effect estimates stratified by timepoint.pdf [file IRNF_A_2698155_SM5762.pdf]

S4 File. Mortality effect estimates stratified by reported mortality time point

Display study label	Original study	No. of studies (K)	Sample size (N)	Effect model	RR	95% CI lower	95% CI upper	p value	I²	EBP modality	Mortality time point	Interpretation	Notes
Chen et al., 2015	Chen et al., 2015	6	642	Fixed-effect	0.99	0.79	1.23	0.92	0	HVHF	28-day mortality	No clear effect	
Kuriyama et al., 2018	Kuriyama et al., 2018	7	307	Fixed-effect	0.76	0.54	1.07	0.006	66.70%	PMX-HP	28-day mortality	No clear effect	
Tian et al., 2020	Tian et al., 2020	7	171	Random-effects	0.80	0.56	1.15	0.001	74.90%	PMX-HP	28-day mortality	No clear effect	
Yin et al., 2020	Yin et al., 2020	3	89		0.96	0.67	1.38	0.48	0%	CRRT; HVHF; [illegible]	28-day mortality	No clear effect	
Snow et al., 2021	Snow et al., 2021	39	1393		0.49	0.37	0.65	<0.001	54%	CVVH; HAD; CPFA; TPE	28-day mortality	Significant reduction	
Wang et al., 2024	Wang et al., 2024	11	1043	Fixed-effect	0.72	0.59	0.88	<0.001	76%	RRT	28-day mortality	Significant reduction	
Li et al., 2024	Li et al., 2024	29	12606	Random-effects	0.75	0.65	0.88	<0.001	63%	PMX-HP	28-day mortality / all-cause mortality	Significant reduction	
Jiovany et al., 2023	Jiovany et al., 2023	17	57		0.98	0.12	8.25	0.13	57%	CytoSorb	28–30-day mortality	No clear effect	
Li et al., 2019	Li et al., 2019	5	184	Random-effects	0.76	0.58	1.00	0.05	76%/86%	CRRT	28-day and 90-day mortality	No clear effect	
Xiao et al., 2022	Xiao et al., 2022	21	1252	Random-effects	0.83	0.70	0.99	0.48	0%	EBP	In-hospital mortality	Significant reduction	
Zhou et al., 2011	Zhou et al., 2011	16	827	Fixed-effect	0.69	0.59	0.80	<0.01		HP + TPE	All-cause mortality	Significant reduction	
Li et al., 2022	Li et al., 2022	5	243		0.75	0.53	1.06	0.02	64%	CPFA	All-cause mortality	No clear effect	
Gong et al., 2015	Gong et al., 2015	4/3	692	Random-effects	0.71	0.38	1.31	0.06	59%	RRT	Overall mortality	No clear effect	
Putzu et al., 2017	Putzu et al., 2017	7	211	Random-effects	0.57	0.36	0.89	0.17	34%	CVVH	Short- and long-term mortality	Significant reduction	
Dinna et al., 2007	Dinna et al., 2007	15	920	Random-effects	0.53	0.43	0.65	0.08	36.30%	PMX-HP	Mortality, time point not specified	Significant reduction	Exact mortality time point was not specified in the extracted summary.
Liu et al., 2010	Liu et al., 2010	4	79	Fixed-effect	0.33	0.17	0.64	<0.01		HVHF	Mortality, time point not specified	Significant reduction	Exact mortality time point was not specified in the extracted summary.
Latour-Pérez et al., 2011	Latour-Pérez et al., 2011	12	505	Random-effects	0.96	0.83	1.12	0.02	52%	CVVH	Mortality, time point not specified	No clear effect	Exact mortality time point was not specified in the extracted summary.
Tian et al., 2012	Tian et al., 2012	5	120	Fixed-effect	0.78	0.57	1.08	0.14	48%	CBP	Mortality, time point not specified	No clear effect	Exact mortality time point was not specified in the extracted summary.
Zhou et al., 2014	Zhou et al., 2014	16	428	Random-effects	0.69	0.56	0.84	<0.01	0.27	HP; PHVHF; CVVH; CVVHDF; [illegible]	Mortality, time point not specified	Significant reduction	Exact mortality time point was not specified in the extracted summary.
Clark et al., 2014	Clark et al., 2014	4	231	Random-effects	0.82	0.52	1.28	0.24	16%	HVHF	Mortality, time point not specified	No clear effect	Exact mortality time point was not specified in the extracted summary.
Rimmer et al., 2014	Rimmer et al., 2014	4	98	Random-effects	0.83	0.45	1.52	0.11	46%	TPE	Mortality, time point not specified	No clear effect	Exact mortality time point was not specified in the extracted summary.
Zhen et al., 2017	Zhen et al., 2017	4	112	Fixed-effect	0.36	0.20	0.66	0.68	0%	HP	Mortality, time point not specified	Significant reduction	Exact mortality time point was not specified in the extracted summary.
Chang et al., 2017	Chang et al., 2017	17		Random-effects	0.81	0.70	0.95	0.04	53.90%	PMX-HP	Mortality, time point not specified	Significant reduction	Exact mortality time point was not specified in the extracted summary.
Terayama et al., 2017	Terayama et al., 2017	7	484		0.65	0.47	0.89	0.002	72%	PMX-HP	Mortality, time point not specified	Significant reduction	Exact mortality time point was not specified in the extracted summary.
Huang et al., 2018	Huang et al., 2018	9	160	Fixed-effect	0.72	0.49	1.07	0.81	0%	PHVHF	Mortality, time point not specified	No clear effect	Exact mortality time point was not specified in the extracted summary.
Putzu et al., 2019 (1)	Putzu et al., 2019	20	1548		0.87	0.78	0.98	0.02	67%	HP	Mortality, time point not specified	Significant reduction	Multiple effect estimates reported for different EBP modalities in the same review. Exact mortality time point was not specified in the extracted summary.
Putzu et al., 2019 (2)	Putzu et al., 2019	13	596		0.79	0.63	1.00	0.05	50%	RRT	Mortality, time point not specified	No clear effect	Multiple effect estimates reported for different EBP modalities in the same review. Exact mortality time point was not specified in the extracted summary.
Putzu et al., 2019 (3)	Putzu et al., 2019	2	128		0.63	0.42	0.96	0.03	0%	TPE	Mortality, time point not specified	Significant reduction	Multiple effect estimates reported for different EBP modalities in the same review. Exact mortality time point was not specified in the extracted summary.
Snow et al., 2019 (1)	Snow et al., 2019	8			0.74	0.28	0.79	0.04	68%	HAD	Mortality, time point not specified	Significant reduction	Multiple effect estimates reported for different EBP modalities in the same review. Exact mortality time point was not specified in the extracted summary.
Snow et al., 2019 (2)	Snow et al., 2019	4			0.81	0.33	2.00	0.65	72%	PMX-HP	Mortality, time point not specified	No clear effect	Multiple effect estimates reported for different EBP modalities in the same review. Exact mortality time point was not specified in the extracted summary.
Zayed et al., 2019	Zayed et al., 2019				0.98	0.85	1.14	0.81		RRT	Mortality, time point not specified	No clear effect	Exact mortality time point was not specified in the extracted summary.
Li et al., 2021	Li et al., 2021	13	599		0.68	0.51	0.91	0.01	78.40%	PMX-HP	Mortality, time point not specified	Significant reduction	Exact mortality time point was not specified in the extracted summary.
Mohammed et al., 2022	Mohammed et al., 2022	5	2165		0.95	0.89	1.07	0.19	35%	RRT	Mortality, time point not specified	No clear effect	Exact mortality time point was not specified in the extracted summary.
Olive et al., 2023	Olive et al., 2023	8	280		0.76	0.61	0.94	0.09	43%	TPE	Mortality, time point not specified	Significant reduction	Exact mortality time point was not specified in the extracted summary.
Yan et al., 2023	Yan et al., 2023	13	688		0.76	0.60	0.97	0.58	0%	BP	Mortality, time point not specified	Significant reduction	Exact mortality time point was not specified in the extracted summary.
Szigetváry et al., 2023	Szigetváry et al., 2023	5			0.64	0.11	3.65	0.52	80%	HAD	Mortality, time point not specified	No clear effect	Exact mortality time point was not specified in the extracted summary.
Zhang et al., 2023	Zhang et al., 2023	5	155	Random-effects	0.54	0.33	0.86	0.009	70%	TPE	Mortality, time point not specified	Significant reduction	Exact mortality time point was not specified in the extracted summary.
Wu et al., 2024	Wu et al., 2024	11	677	Fixed-effect	0.50	0.39	65.00	0.01		HP + HF	Mortality, time point not specified	No clear effect	Exact mortality time point was not specified in the extracted summary.
Hernandez et al., 2024	Hernandez et al., 2024	4	143	Fixed-effect	0.43	0.26	0.72	0.74	0%	TPE	Mortality, time point not specified	Significant reduction	Exact mortality time point was not specified in the extracted summary.
Kuklin et al., 2024	Kuklin et al., 2024	5	166	Random-effects	0.62	0.46	0.83	0.77	0%	TPE	Mortality, time point not specified	Significant reduction	Exact mortality time point was not specified in the extracted summary.
Steindl et al., 2025	Steindl et al., 2025	9	294	Random-effects	0.64	0.42	0.97	0.11	50%	CytoSorb	Mortality, time point not specified	Significant reduction	Exact mortality time point was not specified in the extracted summary.
Orban et al., 2025	Orban et al., 2025	10	355	Random-effects	0.95	0.58	1.56	0.85	57%	CytoSorb	Mortality, time point not specified	No clear effect	Exact mortality time point was not specified in the extracted summary.
