# Supplementary material for: Effects on mortality of different blood purification techniques in sepsis patients: an umbrella review of systematic reviews and meta-analyses
Source: Ren Fail. 2026 Jul 16;48(1):2698155. doi: 10.1080/0886022X.2026.2698155 (PMC13378714; doi:10.1080/0886022X.2026.2698155)
Supplement: S5 File GRADE.pdf [file IRNF_A_2698155_SM5761.pdf]

Outcomes	No.	Risk of bias	Inconsistency	Indirectness	Imprecision	Publication bias	GRADE evidence quality
Mortality	42	High risk of bias	Inconsistency	No indirectness	No imprecision	Publication bias	⨁⨁○○○ Low quality

GRADE domain	Judgment	Rationale
Risk of bias	**Downgraded (–1)**	Many included randomized controlled trials had methodological limitations, including inadequate reporting of randomization, allocation concealment, or blinding. In addition, several meta-analyses did not fully account for trial-level risk of bias when interpreting pooled estimates.
Inconsistency	**Downgraded (–1)**	Substantial statistical heterogeneity was observed across meta-analyses ($I^2 \approx 60\%$ or higher), likely reflecting differences in blood purification modalities, timing of intervention, and patient severity.
Indirectness	**Not downgraded**	The population, intervention, comparator, and outcome directly matched the review question, with no important indirectness identified.
Imprecision	**Not downgraded**	The total sample size was large and the confidence interval did not cross the line of no effect (RR = 1), indicating adequate precision.
Publication bias	**Downgraded (–1, suspected)**	Formal assessments of publication bias were inconsistently reported in the included meta-analyses; therefore, the presence of publication bias could not be excluded.
