# Supplementary material for: Effects on mortality of different blood purification techniques in sepsis patients: an umbrella review of systematic reviews and meta-analyses
Source: Ren Fail. 2026 Jul 16;48(1):2698155. doi: 10.1080/0886022X.2026.2698155 (PMC13378714; doi:10.1080/0886022X.2026.2698155)
Supplement: S4 File Study overlap CCA.pdf [file IRNF_A_2698155_SM5760.pdf]

Metric	Overall including NMAs	irwise-only excluding NM	Meaning
Number of included review sections (c)	42	39	Number of systematic reviews/meta-analyses included in CCA calculation
Raw reference rows extracted	632	550	Raw reference rows after splitting bracket-numbered references
Unique study/reference clusters (r)	419	367	Unique primary-study/reference clusters after DOI/fuzzy-text deduplication
Study/reference occurrences across review	632	550	Number of review-by-reference occurrences after within-review deduplication
Repeated occurrences (N-r)	213	183	Number of repeat occurrences beyond first appearance
Unique clusters appearing in >1 review	90	85	Number of unique clusters that appeared in more than one review
Corrected Covered Area (CCA, %)	1.24	1.31	CCA = (N-r) / [r*(c-1)] * 100
Interpretation	Slight/low overlap (<5%)	Slight/low overlap (<5%)	Common interpretation thresholds: 0–5 slight, 6–10 moderate, 11–15 high, >15 very high

Calculation item	Overall including NMAs	Formula/reference	Pairwise-only excluding NMAs	Formula/reference
c = number of reviews	42	Detected Heading 3 review section	39	Overall c minus 3 NMA sections
r = number of unique clusters	419	COUNT of unique Cluster_ID valu	367	COUNT of unique Cluster_ID values after excluding NMA sections
N = review-reference occurrences	632	COUNT of unique Review + Clust	550	COUNT of unique Review + Cluster_ID pairs after excluding NMA sections
N-r = repeated occurrences	213	213	183	183
CCA	1.2399%	1.2399%	1.3122%	0.013122042
CCA (%)	1.24	1.24	1.31	1.312204216

Review_index	Review	NMA_section	Raw_reference_rows	Unique_reference_clusters_within_review
1	Dinna et al., 2007	No	29	29
2	Liu et al., 2010	No	15	15
3	Latour-Pérez et al., 201	No	12	12
4	Zhou et al., 2011	No	9	9
5	Tian et al., 2012	No	11	11
6	Zhou et al., 2014	No	16	16
7	Clark et al., 2014	No	4	4
8	Rimmer et al., 2014	No	4	4
9	Chen et al., 2015	No	6	6
10	Gong et al., 2015	No	4	4
11	Putzu et al., 2017	No	11	11
12	Zhen et al., 2017	No	6	6
13	Chang et al., 2017	No	17	17
14	Terayama et al., 2017	No	7	7
15	Huang et al., 2018	No	12	12
16	Kuriyama et al., 2018	No	7	7
17	Putzu et al., 2019	No	37	37
18	Li et al., 2019	No	5	5
19	Snow et al., 2019	No	39	39
20	Zayed et al., 2019	No	4	4
21	Tian et al., 2020	No	6	6
22	Yin et al., 2020	No	5	5
23	Li et al., 2021	No	13	13
24	Snow et al., 2021	No	39	39
25	Xiao et al., 2022	No	27	27
26	Li et al., 2022	No	6	6
27	Mohammed et al., 2022	No	5	5
28	Olive et al., 2023	No	14	14
29	Yan et al., 2023	No	13	13
30	Szigetváry et al., 2023	No	28	28
31	Zhang et al., 2023	No	5	5
32	Wu et al., 2024	No	19	19
33	Wang et al., 2024	No	12	12
34	Hernandez et al., 2024	No	6	6
35	Kuklin et al., 2024	No	20	20
36	Li et al., 2024	No	35	35
37	Jiovany et al., 2023	No	6	6
38	Steindl et al., 2025	No	26	26
39	Orban et al., 2025	No	10	10
40	Xing et al., 2025	Yes	45	45
41	Chen et al., 2026	Yes	6	6
42	Meco et al., 2026	Yes	31	31
