# Supplementary material for: Effects on mortality of different blood purification techniques in sepsis patients: an umbrella review of systematic reviews and meta-analyses
Source: Ren Fail. 2026 Jul 16;48(1):2698155. doi: 10.1080/0886022X.2026.2698155 (PMC13378714; doi:10.1080/0886022X.2026.2698155)
Supplement: S2 File Search strategies of eight databases.pdf [file IRNF_A_2698155_SM5759.pdf]

1 知网

Search	Query	Items found
SU%=(脓毒症+脓毒性休克+感染性休克+败血症) AND SU%=(血液净化+血液灌流+血浆吸附+血浆置换+血液透析+血液滤过+肾脏替代治疗+多黏菌素 B+吸附柱+细胞因子清除+CRRT+HVHF+PMX) AND SU%=(meta+荟萃分析+系统评价)		22

2 万方

Search	Query	Items found
题名或关键词:(脓毒症 or 感染性休克 or 脓毒性休克 or 败血症) and 题名或关键词:(血液净化 or 血液灌流 or 血浆吸附 or 血浆置换 or 血液透析 or 血液滤过 or 肾脏替代治疗 or 多黏菌素 B or 吸附柱 or 细胞因子清除 or CRRT OR HVHF OR PMX) and 题名或关键词:(meta or 荟萃分析 or 系统评价)		31

3 维普

Search	Query	Items found
(SU=脓毒症 OR SU=感染性休克 OR SU=脓毒性休克 OR SU=败血症) AND (SU=血液净化 OR SU=血液灌流 OR SU=血浆吸附 OR SU=血浆置换 OR SU=血液透析 OR SU=血液滤过 OR SU=肾脏替代治疗 OR SU=多黏菌素 B OR SU=吸附柱 OR SU=细胞因子清除 OR SU=CRRT OR SU=HVHF OR SU=PMX) AND (SU=meta OR SU=荟萃分析 OR SU=系统评价)		29

4 SinoMed

Search	Query	Items found

("脓毒症"[标题:智能] OR "感染性休克"[标题:智能] OR "脓毒性休克"[标题:智能] OR "败血症"[标题:智能]) AND("血液净化"[标题:智能] OR "血液灌流"[标题:智能] OR "血浆吸附"[标题:智能] OR "血浆置换"[标题:智能] OR "血液透析"[标题:智能] OR "血液滤过"[标题:智能] OR "肾脏替代治疗"[标题:智能] OR "多黏菌素 B"[标题:智能] OR "细胞因子清除"[标题:智能] OR "吸附柱"[标题:智能] OR "CRRT"[标题:智能] OR "PMX"[标题:智能] OR "HVHF"[标题:智能]) AND("meta"[标题:智能] OR "荟萃分析"[标题:智能] OR "系统评价"[标题:智能])	13

5 PubMed

Search	Query	Items found
#1	(("Sepsis"[Mesh]) OR "Shock, Septic"[Mesh])	156,370
#2	(((((((Sepsis*[Title/Abstract]) OR (Septicemia*[Title/Abstract])) OR (Septic shock*[Title/Abstract])) OR (infectious shock[Title/Abstract])) OR (Systemic Inflammatory Response Syndrome[Title/Abstract])) OR (SIRS[Title/Abstract])) OR (septic[Title/Abstract])) OR (septicaemic shock[Title/Abstract])	214,083
#3	#1OR#2	290,619
#4	(((((((((((blood purification[Title/Abstract]) OR (hemoperfusion[Title/Abstract])) OR (plasma adsorption[Title/Abstract])) OR (plasma exchange[Title/Abstract])) OR (plasmapheresis[Title/Abstract])) OR (hemodialysis[Title/Abstract])) OR (hemofiltration[Title/Abstract])) OR ("renal replacement therapy"[Title/Abstract])) OR ("hybrid renal replacement therapy"[Title/Abstract])) OR ("combined blood purification"[Title/Abstract])) OR ("blood purification therapy"[Title/Abstract]))	126,981
#5	((meta-analysis[Title/Abstract]) OR (systematic review[Title/Abstract]))	548,035

#6	#3 AND #4 AND #5	214

6 Embase

Search	Query	Items found
#1	'sepsis'/exp	457,661
#2	'septic shock'/exp	85.344
#3	sepsis*:ti OR septicemia*:ti OR 'septic shock*':ti OR 'infectious shock':ti OR 'systemic inflammatory response syndrome':ti OR sirs:ti OR septic:ti OR 'septicaemic shock':ti	107,409
#4	#1 OR #2 OR #3	458,180
#5	'blood purification':ti OR 'hemoperfusion':ti OR 'plasma adsorption':ti OR 'plasma exchange':ti OR 'plasmapheresis':ti OR 'hemodialysis':ti OR 'hemofiltration':ti OR 'renal replacement therapy':ti OR 'hybrid renal replacement therapy':ti OR 'combined blood purification':ti OR 'blood purification therapy':ti	88,498
#6	'meta-analysis':ti OR 'systematic review':ti	478,616
#7	#4 AND #5 AND #6	100

7 Cochrane Library

Search	Query	Items found
#1	MeSH descriptor: [Sepsis] explode all trees	6,664

#2	MeSH descriptor: [Shock, Septic] explode all trees	1,485
#3	(Sepsis):ti,ab,kw OR (Septicemia):ti,ab,kw OR (Septic shock):ti,ab,kw OR (infectious shock):ti,ab,kw OR (Systemic Inflammatory Response Syndrome):ti,ab,kw OR (SIRS):ti,ab,kw OR (septic):ti,ab,kw OR (septicaemic shock):ti,ab,kw	20,834
#4	#1 OR #2 OR #3	22,288
#5	(blood purification):ti,ab,kw OR (hemoperfusion):ti,ab,kw OR (plasma adsorption):ti,ab,kw OR (plasma exchange):ti,ab,kw OR (plasmapheresis):ti,ab,kw OR (hemodialysis):ti,ab,kw OR (hemofiltration):ti,ab,kw OR (renal replacement therapy):ti,ab,kw OR (hybrid renal replacement therapy):ti,ab,kw OR (combined blood purification):ti,ab,kw OR (blood purification therapy):ti,ab,kw	26,229
#6	(meta-analysis):ti,ab,kw OR (systematic review):ti,ab,kw	31,099
#7	#4 AND #5 AND #6	39

8 Web of science

**Search**	**Query**	**Items found**
#1	Topic:(sepsis OR septicemia OR septic shock OR infectious shock OR systemic inflammatory response syndrome sirs OR septic OR septicaemic shock)	136,894
#2	Topic:(blood purification OR hemoperfusion OR plasma adsorption OR plasma exchange OR plasmapheresis OR hemodialysis OR hemofiltration OR renal replacement therapy OR hybrid renal replacement therapy OR combined blood purification OR blood purification therapy)	171,471

#3	Title:(meta-analysis OR systematic review)	476,116
#4	#1 AND #2 AND #3	247
