# Supplementary material for: Effects on mortality of different blood purification techniques in sepsis patients: an umbrella review of systematic reviews and meta-analyses
Source: Ren Fail. 2026 Jul 16;48(1):2698155. doi: 10.1080/0886022X.2026.2698155 (PMC13378714; doi:10.1080/0886022X.2026.2698155)
Supplement: S6 Effects of blood purification modalities on mortality.docx [file IRNF_A_2698155_SM5758.docx]

**Figure A–G. Effects of blood purification modalities versus control on mortality**

(A) Blood purification (unspecified). (B) CVVH. (C) HAD. (D) HVHF/PHVHF. (E) PMX-HP. (F) RRT. (G) TPE.

Each row represents an included meta-analysis and shows the pooled RR with 95% CI for mortality, comparing blood purification with control. Squares indicate point estimates and horizontal lines indicate 95% CIs; the vertical dashed line denotes no effect (RR=1.0). Analyses are displayed on a logarithmic scale. An asterisk (*) indicates that the 95% CI does not include 1.0. Values left of 1.0 favour blood purification and values right of 1.0 favour control.


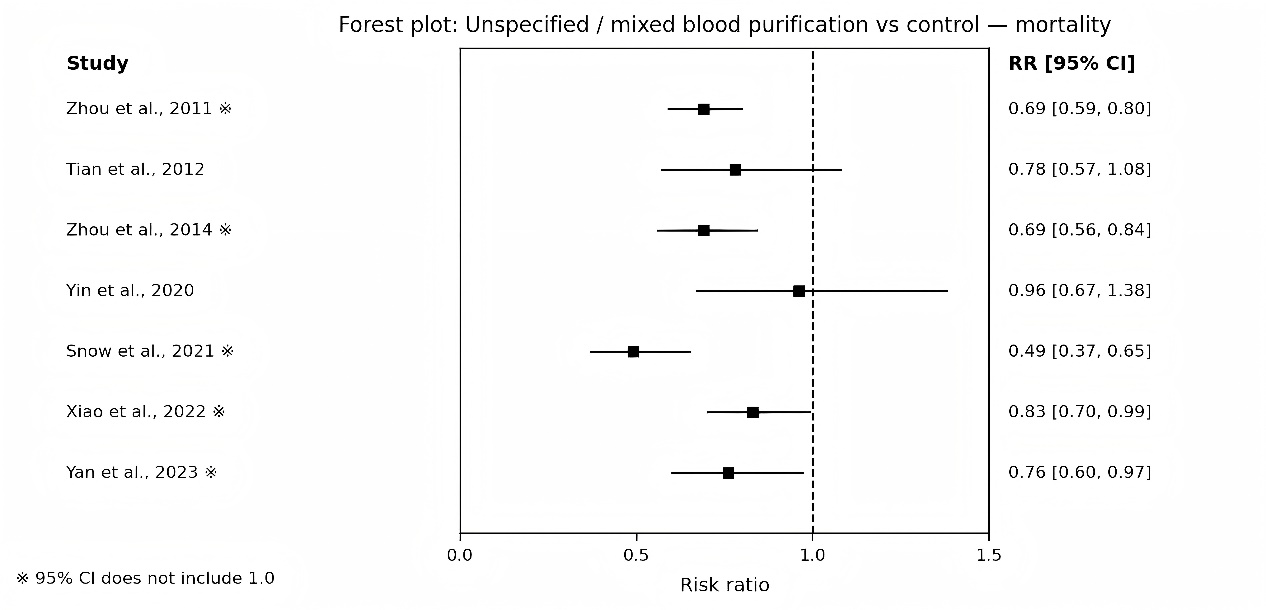


Figure A Blood purification (unspecified)


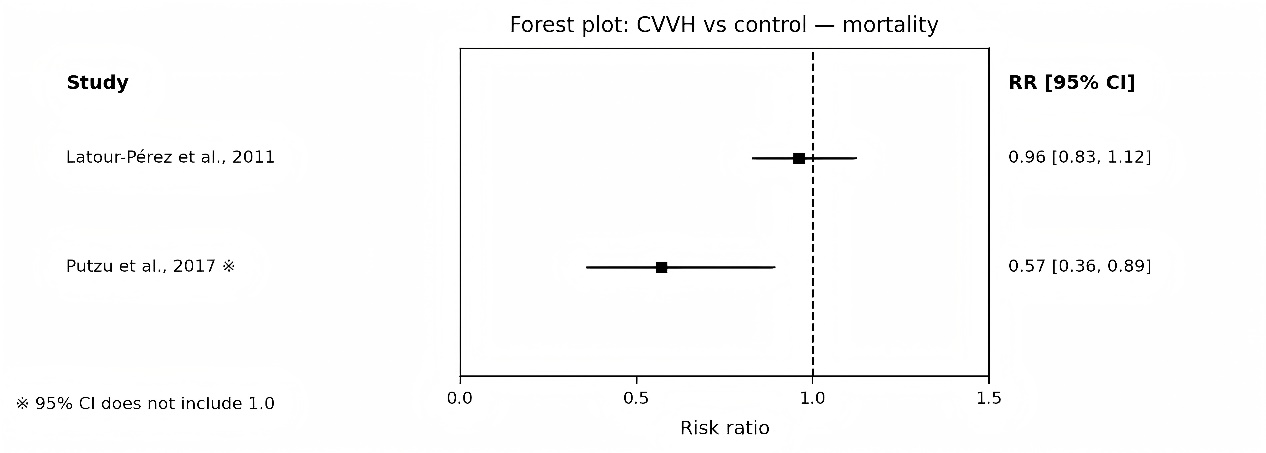


Figure B CVVH


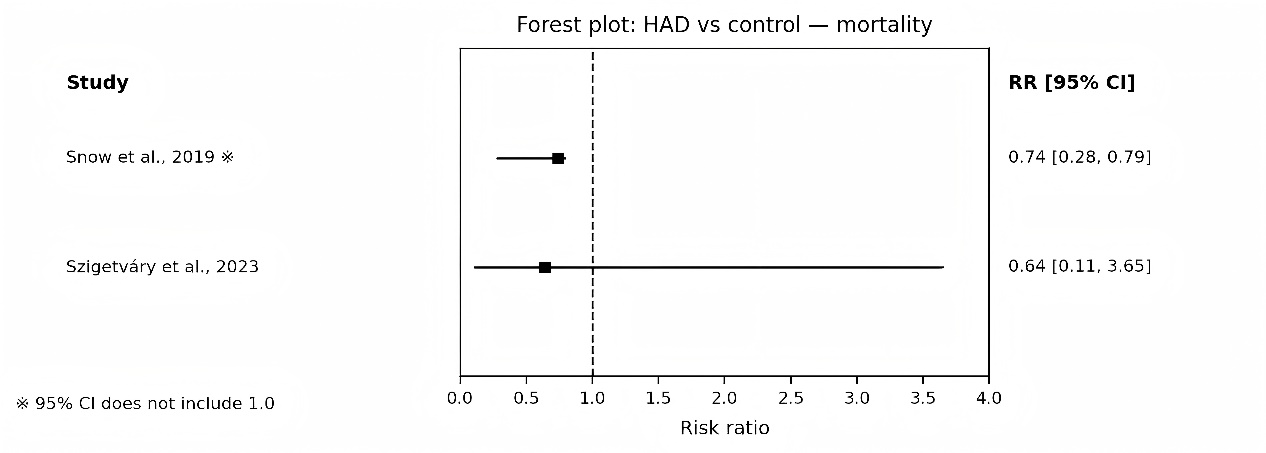


Figure C HAD


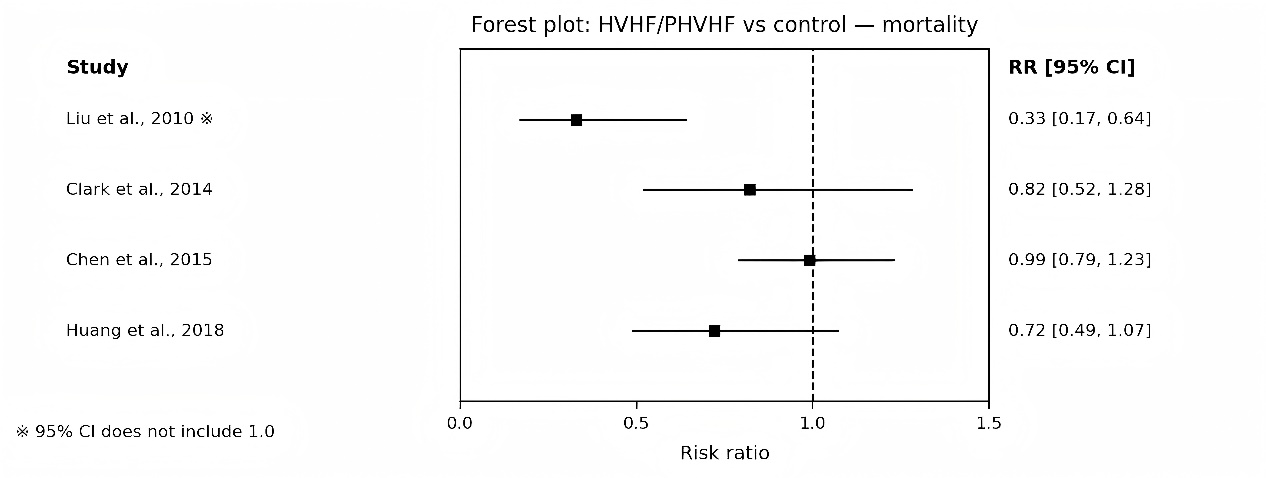


Figure D HVHF/PHVHF


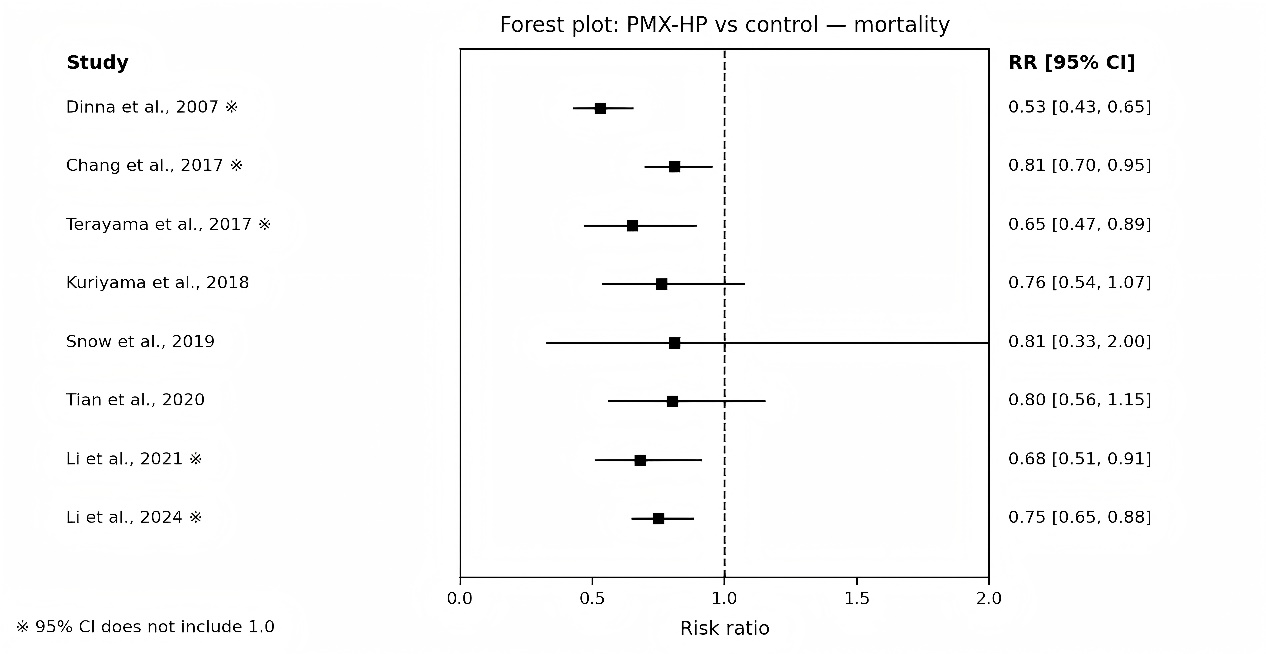


Figure E PMX-HP


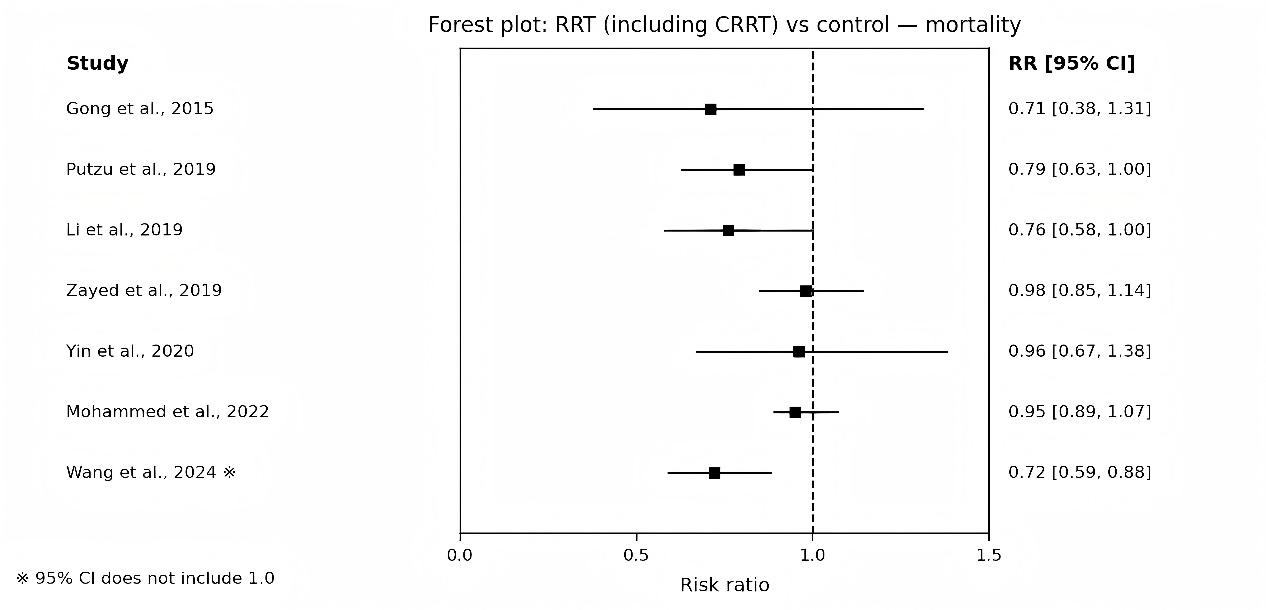


Figure F RRT


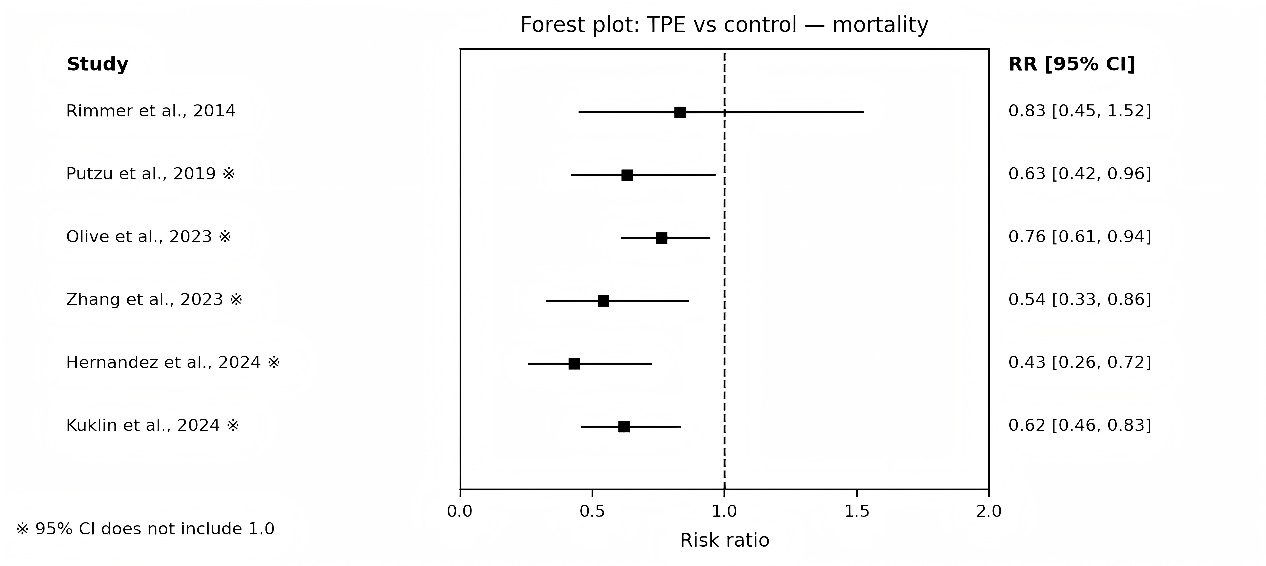


Figure G TPE
